# What can traditional Chinese medicine do for adult neurogenesis?

**DOI:** 10.3389/fnins.2023.1158228

**Published:** 2023-04-12

**Authors:** Wei Shen, Ning Jiang, Wenxia Zhou

**Affiliations:** ^1^School of Chinese Materia Medica, Tianjin University of Traditional Chinese Medicine, Tianjin, China; ^2^Beijing Institute of Pharmacology and Toxicology, Beijing, China; ^3^State Key Laboratory of Toxicology and Medical Countermeasures, Beijing, China

**Keywords:** adult neurogenesis, neural stem cells, traditional Chinese medicine, TCM prescriptions, Chinese herbal medicine, bioactive components

## Abstract

Adult neurogenesis plays a crucial role in cognitive function and mood regulation, while aberrant adult neurogenesis contributes to various neurological and psychiatric diseases. With a better understanding of the significance of adult neurogenesis, the demand for improving adult neurogenesis is increasing. More and more research has shown that traditional Chinese medicine (TCM), including TCM prescriptions (TCMPs), Chinese herbal medicine, and bioactive components, has unique advantages in treating neurological and psychiatric diseases by regulating adult neurogenesis at various stages, including proliferation, differentiation, and maturation. In this review, we summarize the progress of TCM in improving adult neurogenesis and the key possible mechanisms by which TCM may benefit it. Finally, we suggest the possible strategies of TCM to improve adult neurogenesis in the treatment of neuropsychiatric disorders.

## 1. Introduction

Adult neurogenesis is the process of generating functional neurons from neural stem cells (NSCs) (Ming and Song, 2011), which is involved in learning, memory, and emotion and may also be involved in the remodeling of the central nervous system (Taupin, 2005; Lledo et al., 2006; Toda and Gage, 2018). Adult neurogenesis abnormalities play an important role in a variety of neurodegenerative disorders, such as Alzheimer’s disease (AD), Huntington’s disease (HD), and Parkinson’s disease (PD) (Winner and Winkler, 2015; Horgusluoglu et al., 2017; Berger et al., 2020). In addition, adult neurogenesis is associated with emotional illnesses, such as depression (Sahay and Hen, 2007; Vaidya et al., 2007; Berger et al., 2020) and anxiety (Cheung et al., 2016; Toda and Gage, 2018). Stress (Odaka et al., 2017; Schoenfeld et al., 2017) and stroke (Rahman et al., 2021) are also associated with abnormal adult neurogenesis. Considering the role of adult neurogenesis in the pathophysiology of neurological and psychiatric diseases, restoring neurological function by improving adult neurogenesis is one of the main directions in the field of neuroscience.

A lot of work has gone into finding effective medications to boost adult neurogenesis. Recent progress in adult neurogenesis represents a potentially promising target for the treatment of neurological (Taupin, 2008; Matsuda and Nakashima, 2021) and mental conditions (DeCarolis and Eisch, 2010; Jun et al., 2012). Traditional Chinese medicine (TCM) has been used for centuries in China and other Asian countries, such as Korea and Japan. In recent years, TCM, including TCM prescription drugs (TCMPs), Chinese herbal medicine (CHM), and bioactive components extracted from TCM, have been found to have great potential for improving adult neurogenesis in the treatment of neuropsychiatric disorders. In this review, we summarize the effects of TCM on regulating adult neurogenesis and their potential mechanisms and provide the basis for TCM targeting adult neurogenesis in the treatment of neuropsychiatric diseases.

## 2. Adult neurogenesis: From neural stem cells to therapy

### 2.1. Biological significance of adult neurogenesis

**Figure 1:**
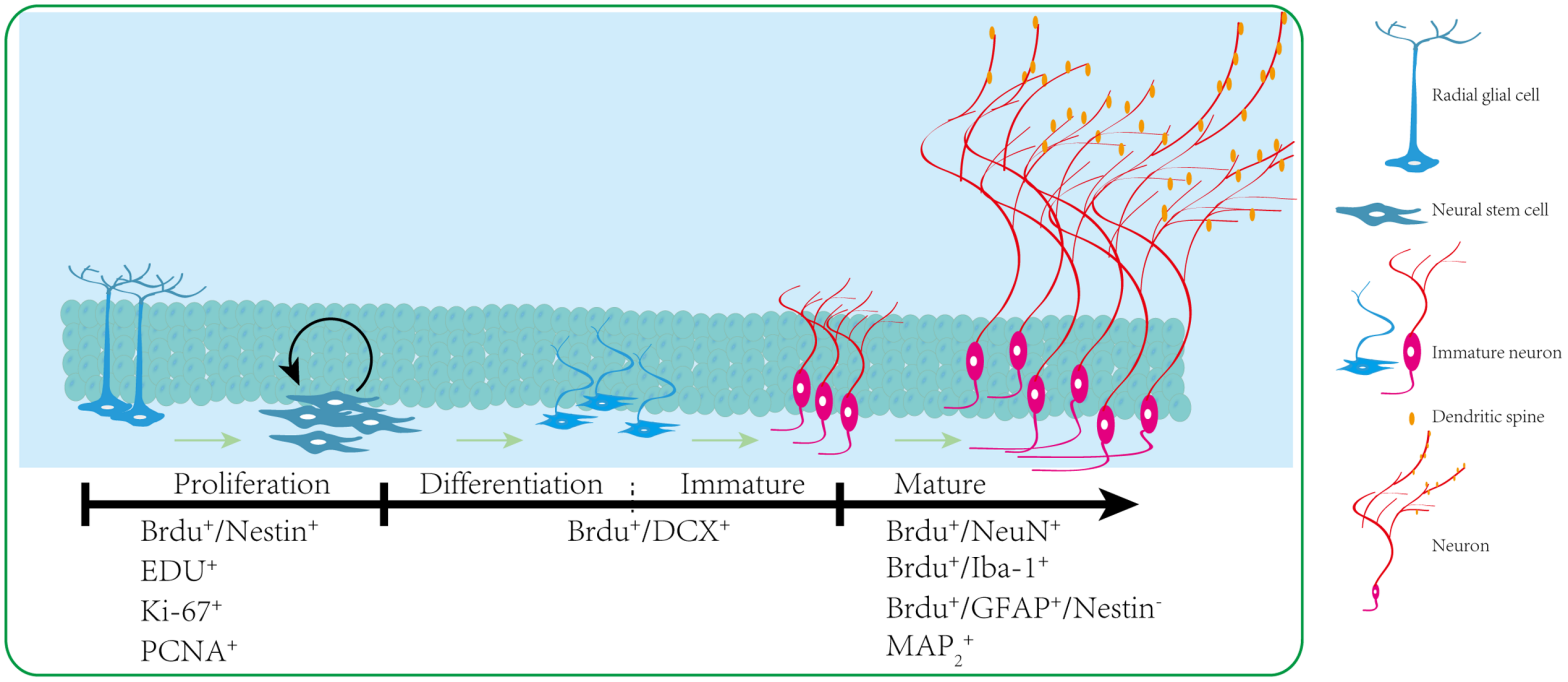
Adult hippocampus of the dentate gyrus. Newly formed neurons in the sub-granular zone of the dentate gyrus pass through several consecutive developmental stages. The radial glial cells can generate proliferating NSCs with transient amplifying characteristics. These NSCs can give rise to neuroblasts that subsequently differentiate into dentate granule neurons. The developmental trajectory is accompanied by the subsequent expression of stage-specific molecular markers.

Neurogenesis is the process by which NSCs proliferate and differentiate to produce new neurons (this process can be seen in Figure 1), which is essential for the development of the brain and the establishment of functional connections. The nervous system of adult mammals has long been considered a non-regenerative tissue. However, in 1965, Altman and Das (Altman and Das, 1965) first observed neurogenesis in adult rats, subsequently, in 1998, Eriksson et al (Eriksson et al, 1998) provided evidence for the existence of adult neurogenesis of human. Over the next decade, the evidence for adult human neurogenesis has been refined (Boldrini et al., 2018; Sorrells et al., 2018; Moreno-Jimenez et al., 2021), confirming that adult neurogenesis exists throughout life (Zhou et al., 2022). With the deepening of adult neurogenesis research, it has been confirmed that adult neurogenesis occurs in two regions of the adult brain: the subgranular zone of the hippocampus (SGZ) and the subventricular zone (SVZ) of the lateral ventricles of adult mammals (Gould, 2007).

There is growing evidence that adult neurogenesis is essential for central nervous system (CNS) function. Adult neurogenesis is associated with cognition and emotion (Anacker and Hen, 2017; Alam et al., 2018). Adult neurogenesis is involved in cognition, including memory interference and indexing (Miller and Sahay, 2019), learning (Yau et al., 2015), and forgetting (Akers et al., 2014). Adult neurogenesis is also involved in the regulation of mood (Anacker and Hen, 2017), reduced neurogenesis has been implicated in the pathogenesis of anxiety and depression (Snyder et al., 2011), and increasing adult neurogenesis is sufficient to reduce anxiety and depression-like behaviors (Hill et al., 2015). Meanwhile, researchers have found that adult neurogenesis confers stress resilience (Anacker et al., 2018), and this resilience is necessary for the body to adapt to new environments. In addition, after a brain injury, the new neurons generated by adult neurogenesis are essential for the recovery of neural function (Marques et al., 2019).

It is known that NSCs progress through distinct stages before they become mature neurons, and this process is tightly controlled by cell-intrinsic factors and signals in the neurogenic niche (Johnson et al., 2009; Suh et al., 2009). In short, adult neurogenesis is tightly regulated by cell-intrinsic molecules and extrinsic signaling. Intrinsic signaling involves phosphoinositide 3-kinase (PI3K)/Akt, Notch-Hairy, and enhancer of split (Notch-Hes) signaling, Hedgehog signaling, bone morphogenetic protein signaling, and Wingless/Integrated signaling (Goncalves et al., 2016; Matsubara et al., 2021). Extracellular signaling is mainly from the NSC niche that creates a favorable microenvironment and architecture to sustain NSCs and neurogenesis (Li and Guo, 2021). Such factors as growth factors, neurotrophic factors, and neurotransmitters have also been reported to be part of the regulatory signaling within the hippocampal niche (Goncalves et al., 2016). Importantly, intrinsic and extrinsic signaling crosstalk and act on the CNS to regulate neurogenesis.

As its existence has been questioned in the past, studies have sought to understand how adult neurogenesis affects the human brain in both health and disease. Researchers have also looked at the factors that may affect this process. The above developments greatly promote our understanding of adult neurogenesis and how it might be used to enhance CNS performance and for the prevention and treatment of diseases that affect it.

### 2.2. Adult neurogenesis and neuropsychiatric diseases

With the growing understanding of the role of adult neurogenesis in the regulation of cognitive function, emotion, and brain repair after injury, the study of the relationship between this process and neuropsychiatric diseases has also made progress. Changes in adult neurogenesis were observed in neurological (such as AD, PD, HD, and stroke) and psychiatric (depression and post-stroke depression) diseases, and adult neurogenesis has been found to be involved in the pathological mechanisms of these diseases. Improving adult neurogenesis has been tried as a means of alleviating neurological and psychiatric disorders.

Alterations in adult neurogenesis have been reported in most neurological disorders, including neurodegenerative diseases and stroke. Since adult neurogenesis is involved in the regulation of cognition (Anacker and Hen, 2017), modulating adult neurogenesis may help to improve cognitive deficits in some neuropsychiatric disorders (Berger et al., 2020). In fact, abnormal adult neurogenesis has been observed in neurodegenerative diseases such as AD, HD, PD, and amyotrophic lateral sclerosis (ALS) (Jordan et al., 2006; Vivar, 2015; Horgusluoglu et al., 2017), which manifest as cognitive decline (Winner and Winkler, 2015; Terreros-Roncal et al., 2021). In contrast to the reduction of neurogenesis in neurodegenerative diseases, the proliferation of NSCs and the production of neuroblasts were activated after stroke. These neuroblasts migrate to the infarcted area, contribute to the repair of the infarcted brain, and form glial scar tissue (Koh and Park, 2017). However, based on comparisons between the density of BrdU-stained cells colabeled with a neuronal marker at 2 and 6 weeks post-ischemia about 80% or more of the new neurons died during this time interval (Arvidsson et al., 2002). Meanwhile, the effect of compensatory neurogenesis in repairing and restoring neural function has been limited. Fortunately, exogenous transplantation of NSCs (Hassani et al., 2012) and drugs (Chen et al., 2016) can be beneficial for neurogenesis and contribute to the recovery of brain function (such as motor balance and cognition) after stroke (Jin et al., 2006), and promoting adult neurogenesis has become an important direction for post-stroke recovery treatment (Marques et al., 2019).

Alterations in adult neurogenesis and reduced size of the hippocampus were reported in most psychiatric disorders, including schizophrenia, major depression, addiction, and anxiety, and in a significant subpopulation of patients with depression (Goncalves et al., 2016). Depressive disorders may be caused by impaired adult hippocampal neurogenesis in adults (Miller and Hen, 2015), and the effects of antidepressants have been found to relate to neurogenesis (Santarelli et al., 2003). Currently, medicine uses antidepressants such as fluoxetine, sertraline, and paroxetine, which could improve impaired cognitive, emotional, and motor function by promoting adult neurogenesis (Li et al., 2009).

However, to date, there isno clinical evidence of an isolated impairment of adult hippocampal neurogenesis in the absence of other abnormalities,but numerous studies have reported alterations in adult neurogenesis that are associated with several neurological and psychiatric disorders, providing a link between adult neurogenesis and human disease (Goncalves et al., 2016).

Since neurogenesis is related to a variety of neurological and psychiatric diseases, researchers have begun to try to alleviate diseases by influencing neurogenesis and have made some progress. The amelioration of diseases by neurogenesis mainly includes intracerebral transplantation and endogenous activation of NSCs (Chrostek et al., 2019; Wang J. et al., 2021). Although clinical data or evidence of a causal relationship between adult neurogenesis and disease are still lacking, a growing body of evidence in rodents and non-human primates indicates that improving adult neurogenesis contributes to restoring brain function in neuropsychiatric disorders. On this basis, research was carried out on the treatment of diseases with NSC transplantation or endogenous activation of NSCs. NSC transplantation could improve AD, PD, depression (Bao and Song, 2018), stroke, and other diseases (Boese et al., 2018). Promoting adult neurogenesis through endogenous activation of NSCs may also have good application prospects through lifestyle interventions or drugs. In lifestyle practice, exercise, environmental enrichment, and even dietary factors have been shown to enhance adult neurogenesis in animal models and can effectively alleviate depression and cognitive decline associated with animal models of mental illness (Hueston et al., 2017; Ma C. L. et al., 2017; Gronska-Peski et al., 2021). Adult neurogenesis is improved by medication for the symptoms of depression (Elder et al., 2006; Zeng et al., 2022), AD (Ye et al., 2016; Stazi and Wirths, 2021), and stroke (Chen et al., 2016), with TCM having the greatest effects on the aforementioned adult neurogenesis-related diseases. Together, these findings show that improving adult neurogenesis is indeed one of the most important ways to treat diseases. TCM has a variety of clinical procedures to treat neurological and psychiatric diseases and brain injuries, and these procedures have a proven track record of success. Improving adult neurogenesis may also be one of the key mechanisms underlying these procedures’ efficacy.

## 3. The effects of TCM on adult neurogenesis in neurological and psychiatric diseases

TCM has good clinical effects in the treatment of CNS diseases. Some researchers suggest that adult neurogenesis may be the mechanism of TCM for CNS diseases (Ren and Zuo, 2012; Yang et al., 2017; Wang J. et al., 2021; Feng et al., 2022). Thus, TCM has great potential for targeting adult neurogenesis to improve CNS diseases. Indeed, it has been observed that TCM prescriptions (TCMPs), CHMs, and bioactive components derived from TCM could affect adult neurogenesis and improve cognition, alleviate mood, and restore brain function in the animal model. In addition, different TCM may be involved in the regulation of different stages of adult neurogenesis.

### 3.1. The effects of TCM prescriptions on adult neurogenesis

**Figure 2:**
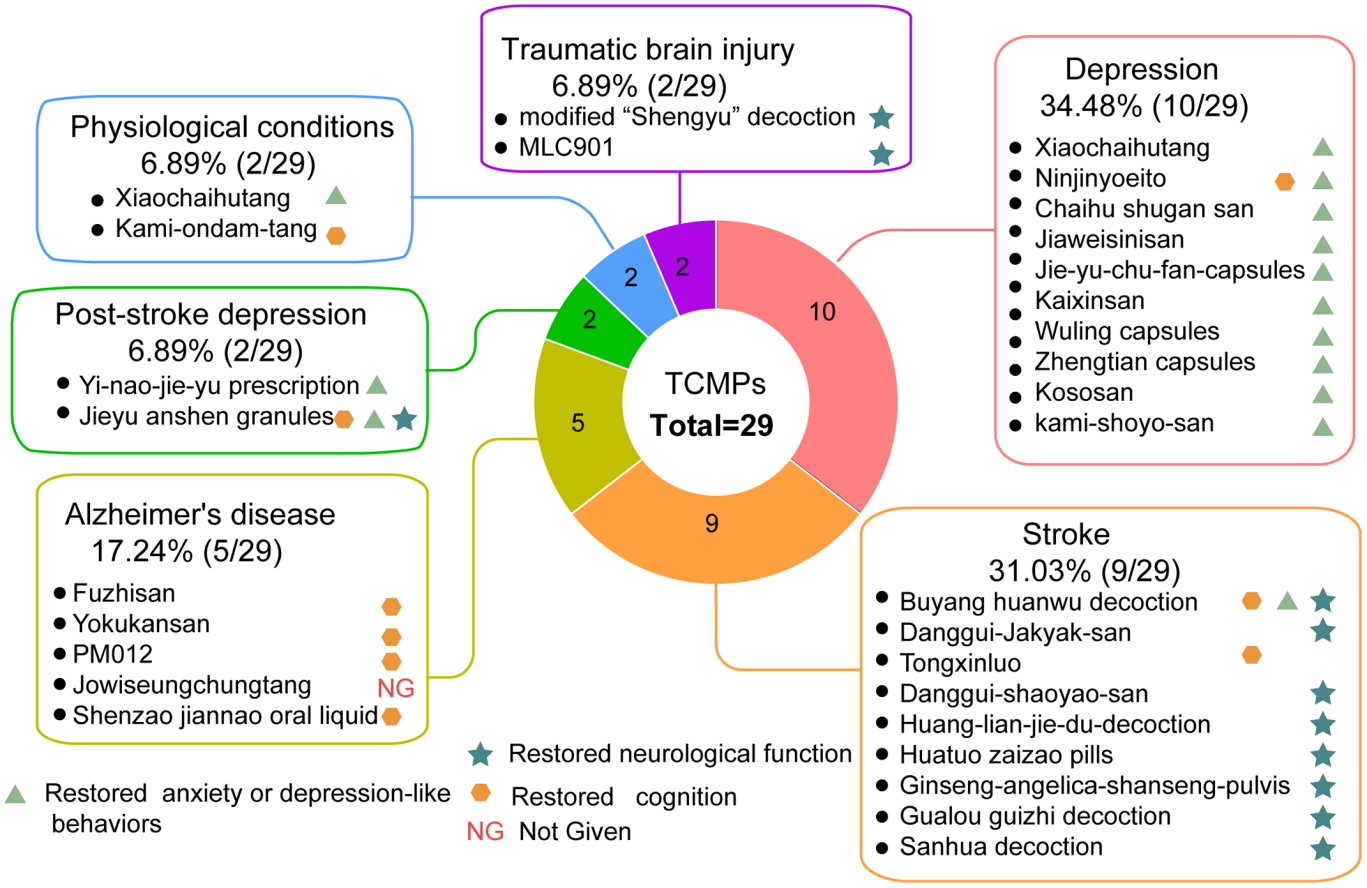
Pie chart of TCMPs improving adult neurogenesis and brain function, *n* = 29. Xiaochaihutang improves adult neurogenesis in stroke and physiological conditions, so it was counted in two cases for two situations.

In recent years, more and more researchers have focused on TCM’s improvement of CNS diseases by targeting adult neurogenesis. Table 1 and Figure 2 show that 28 kinds of TCMPs were reported to improve abnormal adult neurogenesis, which may be related to neurological and psychiatric diseases. Besides, two kinds of TCMPs were also reported to improve adult neurogenesis under normal physiological conditions.

**Table 1:** Effects of TCM prescriptions on neurological conditions and adult neurogenesis.

Prescription	Composition	Animals/cell types	Outcome measurement	Aspects of behaviors/function	References
Buyang Huanwu decoction	Hedysari Radix, Angelicae Sinensis Radix, Paeoniae Rubra Radix, Chuanxiong Rhizoma, Persicae Semen, Carthami Flos, Pheretima.	Male ICR mice were subjected to an acute ischemic stroke by inducing a middle cerebral ischemic/reperfusion (CIR) injury	cerebral cortex:↑MAP-2^+^/BrdU^+^ at day 7 and day 14 after stroke.	↑ Brain function, ameliorated the cerebral infarction, and significantly improved the neurological deficits	Wang et al. (2011)
		A rat model of cerebral ischemia by MCAO	Cerebral cortex, SGZ and SVZ:↑BrdU^+^/MAP2^+^	Not given	Liu et al. (2013)
		cerebral ischemia/reperfusion (CIR) injury ICR mouse model	SGZ and SVZ: ↑DCX^+^	↑Locomotor activity and behavior response in a novel open field	Chen H. J. et al. (2015)
		Adult male Sprague–Dawley rats MCAO ischemic	DG: ↑DCX^+^ ↑GFAP/BrdU-positive cells	↑Learning function but not memory functions by Water maze test	Chen et al. (2020)
		C17.2 neural stem cells	↑BrdU^+^, nestin^+^ in the NSCs ↑Tuj1^+^ and GFAP^+^	Cell	Chen et al. (2020)
		cerebral ischemia/reperfusion (CIR) injury	cerebral cortex: ↑BrdU^+^/DCX ^+^, BrdU^+^/NeuN^+^	↑Modified neurological severity score (mNSS) and the corner test	Zhuge et al. (2020)
Danggui-Jakyak-San	Paeoniae Radix, Atractylodis Rhizoma, Alismatis Rhizoma, Hoelen, Cnidii Rhizoma, and Angelicae Gigantis Radix.	Male C57BL/6 mice (22–26 g, 7 weeks) bilateral common carotid artery occluded ischemia (BCCAO)	DG: ↑Ki67, DCX^+^, BrdU^+^, ↑BrdU^+^/ NeuN^+^; ↑BrdU^+^/DCX^+^, BrdU^+^ /GFAP^+^	↑ Spatial memory in the Morris water maze	Song et al. (2013)
Tongxinluo	Ginseng Radix et Rhizoma, Hirudo，Scorpio, Paeoniae Radix Rubra, Cicadae Periostracum，Eupolyphaga Steleophaga, Scolopendra, Santali Albi Lignum, Dalbergiae Odoriferae Lignum, Olibanum，Ziziphi Spinosae Semen, Borneolum.	male Sprague–Dawley rats receive permanent distal middle cerebral artery occlusion (MCAO)	ipsilateral thalamus:7 days: ↑BrdU^+^, Nestin+14 days: ↑BrdU^+^, Nestin^+^, BrdU^+^/ Nestin^+^, BrdU^+^/NeuN^+^	↑Neurological function (Bederson scores) without reducing infarction volume (Nissl staining)	Chen L. et al. (2014)
		The MCAO model in the hypertensive rats	SVZ: ↑BrdU^+^/NeuN^+^ cells, BrdU^+^ /DCX^+^	↑Neurological Function (Bederson scores)	Chen et al. (2016)
Danggui-Shaoyao-San	Angelicae Sinensis Radix, Paeoniae Radix Alba, Smilacis Glabrae Rhizoma, Atractylodis Macrocephalae Rhizoma, Alismatis Rhizoma, Chuanxiong Rhizoma.	Female Sprague–Dawley rats MCAO was induced by intraluminal occlusion for 90 min with a nylon monofilament suture	SVZ: ↑ DCX^+^, BrdU^+^/DCX^+^	Improved Neurological deficits (body posture and sensorimotor integration) motor deficits also improved based on The elevated body swing test	Ren et al. (2015)
Huang-Lian-Jie-Du-Decoction (HLJDD)	Coptidis Rhizoma, Scutellariae Radix, Phellodendri Chinensis Cortex, Gardeniae Fructus.	Male Sprague–Dawley rats, create the permanent middle cerebral artery occlusion (pMACO)	ipsilateral cortex:Alkaloids: ↑BrdU^+^,BrdU^+^/MAP2^+^ iridoids:↑BrdU^+^,BrdU^+^/MAP2^+^ flavonoids:↑BrdU^+^/MAP2^+^、↓BrdU^+^/GFAP^+^	↑Bederson scores and motor coordination (Beam walking test)	Zou et al. (2016)
Huatuo Zaizao pill	Chuanxiong Rhizoma, Borneol, Euodiae Fructus, Carthami Flos, angelicae Sinensis Radix.	Male Sprague–Dawley rats with Cerebral I/R model	peri-infarct regions of cortex of rats:↑ EdU^+^ /NeuN^+^	↑Cylinder test (assessed forelimb use asymmetry) Beam-walking test (coordination and integration of motor movements) and Adhesive (assess the sensorimotor deficit)	Duan et al. (2017)
Ginseng-Angelica-Shanseng-Pulvis (GASP)	Ginseng Radix et Rhizoma, Angelicae Sinensis Radix, Cinnamomi Cortex.	Male Sprague–Dawley (SD) rats with permanent MCAO	SVZ: 4.6 or 9.2 g/kg:↑Ki67^+^ SGZ:2.3 g/kg:↓DCX^+^, ↑DCX^+^/NeuN^+^, GFAP^+^, Nestin^+^ 4.6 or 9.2 g/kg:↑DCX^+^, DCX^+^/NeuN^+^, GFAP^+^, Nestin^+^	↑Sensorimotor functions (Basket Test and Adhesive Removal Test) and Recognition Memory (novel object recognition test); Cerebral Blood Flow and Infarction Volume	Liu et al. (2019)
Gualou Guizhi decoction	Trichosanthis Radix, Cinnamomi Ramulus, Paeoniae Radix Alba, Glycyrrhizae Radix, Zingiberis Rhizoma Recens, Jujubae Fructus.	Sprague Dawley rats；Transient MCAO surgery	SVZ: ↑BrdU^+^, DCX^+^,BrdU^+^/DCX^+^, Striatum: ↑BrdU^+^/GFAP^+^	↓ The modified neurological severity score and the balance beam score a lower percentage of foot faults	Han et al. (2018)
Sanhua Decoction (SHD)	Rhei Radix et Rhizoma, Notopterygii Rhizoma et Radix, Magnoliae Officinalis Cortex, Aurantii Fructus Immaturus.	Sprague–Dawley (SD) rats; MCAO	SVZ: ↑BrdU^+^,BrdU^+^/DCX^+^	↓ Neurological Deficit Scores	Fu et al. (2020)
Yi-nao-jie-yu prescription (YNJYP)	Acanthopanacis Senticosi Radix et Rhizoma Seu Cauls, Curcumae Radix, Schisandrae Chinensis Fructus, Gardeniae Fructus, Salviae Miltiorrhizae Radix Et Rhizoma, Chuanxiong Rhizoma.	Sprague–Dawley rats; Combined MCAO and Depression Model	DG: ↑BrdU^+^ /NeuN^+^ DG: ↓BrdU^+^/GFAP^+^	↓ The immobility time of forced swim test ↑ increased the sucrose preference	Tian et al. (2018)
Jieyu Anshen granule (JY)	*Bupleuri Radix, Jujubae Fructus, Acori Tatarinowii* Rhizoma, Pinelliae Rhizoma Praeparatum *Cum* Zingibere et Alumine, Atractylodis Macrocephalae Rhizoma, Tritici Levis Fructus, Polygalae Radix, Glycyrrhizae Radix et Rhizoma, Gardeniae Fructus, Lilii Bulbus, Arisaema *Cum* Bile, Curcumae Radix, Dragon’s Teeth, Ziziphi Spinosae Semen, Poria, Angelicae Sinensis Radix.	Sprague–Dawley rats:MCAO + CUMS (MCAO, then CUMS for 18 days	DG: ↑ BrdU^+^/NeuN^+^	↑Open-field and sucrose preference tests, in beam-walking, cylinder, grip strength, and water maze tests	Du et al. (2020)
modified “Shengyu” decoction (MSD)	Rehmanniae Radix Praeparata, Paeoniae Radix Alba, Chuanxiong Rhizoma, Ginseng Radix et Rhizoma, Angelicae Sinensis Radix, Salviae Miltiorrhizae Radix et Rhizoma, Astragali Radix, Myrrha, Acori Tatarinowii Rhizoma, Curcumae Radix.	Sprague–Dawley rats with TBI	Cortex, CA1, CA3,and DG:↑BrdU^+^/Nestin^+^	↑Neurological functions by beam balance and prehensile traction tests	Chen M. M. et al. (2015)
MLC901	Astragali Radix, Salvia Miltiorrhizae Radix, Paeoniae Radix Rubra, Chuanxiong Rhizoma, Angelicae Sinensis Radix, Carthami Flos, Persica Prunus, Polygalae Radix, Acori Tatarinowii Rhizoma.	Male Sprague–Dawley rats with TBI	DG: ↑BrdU^+^	↑Modified version of object recognition task called the “what-where-when” test	Quintard et al. (2014)
Kami-ondam-tang	Pinelliae Rhizoma, Bambusae Caulis, Aurantii Immaturus Fructus, Poria, Citri Reticulatae Pericarpium, Glycyrrhizae Radix, Polygalae Radix, Scrophulariae Radix, Ginseng Radix, Rehmanniae Radix, Zizyphi Spinosae Semen, Jujubae Fructus, Zingiberis Rhizoma.	Male ICR mice	DG: ↑ DCX^+^	↑Step through latency in the retention trial of the passive avoidance task	Hong et al. (2011)
Xiaochaihutang	Bupleuri Radix, Scutellariae Radix, Ginseng, Radix Glycyrrhizae, Zingiberis Rhizoma Recens, Jujubae Fructus.	Kunming mice	DG(HRG): ↑Ki-67^+^,DCX^+^	↓Immobility duration in Tail suspension test and Forced Swim. the latency in Novelty Suppressed Feeding Test ↑ immobility latency in Forced Swim Test.	Zhang et al. (2015)
		Mice were injected subcutaneously with CORT (40 mg/kg) dissolved in sesame oil for 35 days.	DG:XCHT (2.3,7,21 g/kg):↑Ki-67 ^+^ XCHT (7,21 g/kg):↑Ki-67^+^, DCX^+^	↑Weight, the coat state, the escape behavior in open field test and elevated plus maze, immobility time in tail suspension test and forced swimming test.	Zhang et al. (2016)
		Male C57 BL/6 J mice were reared isolated for 8 weeks	DG:XCHT (2.3 g/kg):↑Ki-67 ^+^ XCHT (7.0 g/kg):↑Ki-67^+^, BrdU^+^, DCX^+^	↑ Immobility time in TST and FST, OFT and EPM, aggressive behaviors of SI-reared mice.	Ma C. L. et al. (2017) and Ma J. et al. (2017)
Chaihu Shugan San	Bupleuri Radix, Citri Reticulatae Pericarpium, Chuanxiong Rhizoma, Cyperi Rhizoma, Aurantii Fructus, Paeoniae Radix Alba, Glycyrrhizae Radix et Rhizoma.	perimenopausal rats exposed to chronic unpredictable mild stress (CUMS).	DG:2 g/kg CSS:↑ DCX^+^	↑ The sucrose preference. ↓immobility time of the forced swimming test.	Chen et al. (2018)
		C57BL/6 mice exposed to chronic unpredictable mild stress (CUMS).	DG: ↑BrdU^+^, NeuN^+^/BrdU^+^	↑Sucrose preference ↓immobility time in the TST and FST	Zhang et al. (2021)
Kaixinsan	Ginseng Radix et Rhizoma, Smilacis Glabrae Rhizoma, Polygalae Radix, Acori Tatarinowii Rhizoma.	Cortical and hippocampal neurons, from SD rat embryos at days of 18	KXS2012 in DIV 5 of cortical neurons: ↑synaptic vesicle protein, synaptotagmin KXS2012 in DIV 15 of cortical neurons: ↑the dendritic spine density;↑synaptotagmin expression	↑Sucrose preference; cumulative immobility time of forced swimming test; open field tests	Yan et al. (2016)
		male Sprague–Dawley rats；CMS rat models of depression	Functional analysis: differentially expressed proteins participate in synaptic plasticity, neurodevelopment, and neurogenesis	↑Sucrose consumption and body weight	Dong et al. (2020)
kami-shoyo-san	Paeoniae Radix; Bupleuri Radix; Atractylodis Macrocephalae Rhizoma; Liriopis Tuber; Angelicae Gigantis Radix; Hoelen; Menthae Folium; Glycyrrhizae Radix; Zingiberis Rhizome.	Sprague–Dawley rats; Immobilization stress for 21 days (Stress group)	DG (KSS 20X):↑ BrdU^+^	↓Immobility times compared to the control group.	Park et al. (2007)
Jiaweisinisan	Bupleurum, Peony Root, Citrus Aurantium, Medlar, Gardenia, Rehmanniae, Abalone.	a stress damage model was established with 120 μM corticosterone	↑BrdU^+^ ↓BrdU^+^/TUNEL^+^	Cell	Wu et al. (2013)
		Wistar rats weighing;6-week Chronic Unpredictable Mild Stress (CUMS) model	DG: ↑BrdU^+^/DCX^+^	↑Sucrose preference, locomotion activity level and accuracy of T-maze, as well as increased immobility time	Wang H. Z. et al. (2021) and Wang J. et al. (2021)
Wuling Capsule	*Wuling*	Sprague–Dawley rats were subjected to3-week CMS to induce depression	DG: ↑ BrdU^+^	↑Sucrose preference	Li et al. (2010)
Kososan	Cyperi Rhizoma, Perillae Herba, Aurantii Nobilis Pericarpium, Glycyrrhizae Radix, Zingiberis Rhizoma	Male C57BL/6 J were exposed to 10 min of social defeat stress from an aggressive CD-1 mouse for 10 consecutive days (days 1–10).	DG: ↑BrdU^+^/DCX^+^	↑Social avoidance, depression- and anxiety-like behaviors,	Ito et al. (2017)
Ninjinyoeito	Rehmannia Root, Japanese Angelica Root, Atractylodes Rhizome, Poria Sclerotium, Ginseng, Cinnamon Bark, Polygala Root, Peony Root, Citrus Unshiu Peel, Astragalus Root, Glycyrrhiza, Schisandra Fruit.	C57BL6 mice were administered CORT (100 mg/m) in place of drinking water for 14 days ， Animal were weaned with 50 mg/ml CORT for 3 days and then with 25 mg/ml CORT for 3 days to allow for gradual recovery of endogenous corticosterone secretion.	DG: ↑Ki67, DCX^+^	↓ Immobility and latency to immobility of the tail suspension; ↑ the latency to immobility of the forced swim test; sucrose consumption rate; spontaneous alternations with Y-maze test; spent more time with the novel object	Murata et al. (2018)
		NPCs from the adult mouse hippocampus;NPCs were cultured for 72 h in the presence of 20 mM CORT	↑BrdU^+^ in a dose dependent manner	Cell	Murata et al. (2018)
Jie Yu Chu Fan (JYCF) capsule	Gardeniae Fructus, Magnoliae Officinalis Cortex, Pinelliae Rhizome, Forsythiae Fructus.	C57BL/6 mice were subjected to the following mild stressors for 5 weeks (CUMS)	DG: ↑Ki-67^+^, NeuN^+^, MAP-2^+^	↑Weight; the number of crossings of open field test; sucrose preference; ↓ immobility time of the forced swim test	Ji et al. (2020)
Zhengtian capsule (ZTC)	Spatholobi Caulis, Angelicae Sinensis Radix, Chuanxiong Rhizoma, Asari Radix Et Rhizoma, Uncariae Ramulus *Cum* Uncis, Paeoniae Radix Alba, Angelica Dahuricae Radix, Rehmanniae Radix, Saposhnikoviae Radix, Notopterygii Rhizoma et Radix, Persicae Semen, Carthami Flos, Angelicae Pubescentis Radix, Ephedrae Herba, Aconiti Lateralis Radix Praeparata,	Kunming (KM) mice were intraperitoneally injected with a single dose of LPS (5 mg/kg)	DG: ↑BrdU^+^, ↑GAD67^+^, DCX^+^, BrdU^+^/DCX^+^	↑The crossing numbers and the grooming numbers; coordination and balance of exercise	Yang et al. (2020)
Fuzhisan	Ginseng, Baical, Acorus Talarinowi Rhizoma, Glycyrrhizae Radix.	Eight-month-old male SAMP-8	SGZ: ↑BrdU^+^, PCAN^+^	↓The average escape latency, ↑The number of crossings of the platform location	Yang et al. (2011)
Yokukansan (YKS)	Atractylodes Lancea Rhizome, Poria Sclerotium, Cnidium Rhizome, Angelica Radix, Uncaria Uncis *Cum* Ramulus, Bupleurum Radix, Glycyrrhizae Radix	Male SAMP8 mice at 5 months of age	DG: ↑ BrdU^+^	↓The escape latency and the swimming path length	Azuma et al. (2018)
Herbal formula PM012	Lycii Fructus, Rehmanniae Radix, Corni Fructus, Dioscoreae Radix, Hoelen, Alismatis Radix, Mountain Cortex Radices.	3xTg mice carrying a mutant APP (KM670/671NL), a human mutant PS1 (M146V) knock-in and tau (P301L) transgenes [B6;129-Psen1tm1Mpm Tg(APPSwe,tauP301L)1Lfa/J] mice	CA1 and DG: PM012 (100 mg): ↑BrdU^+^/NeuN^+^. PM012 (400 mg):↑DCX^+^, BrdU^+^/NeuN^+^	↓ Escape latencies， increased time spent in the target zone during probe tests.	Ye et al. (2016)
Jowiseungchungtang (JWS)	Coicis Semen, Castaneae Semen, Raphani Semen, Longanae Arillus, Liriopis Tuber, Platycodi Radix, Acori Gramineri Rhizoma, Thujae Semen, Zizyphi Semen, Massa Medicata Fermentata, Ephedrae Herba, Schisandrae Fructus, Amomi Semen, Polygalae Radix.	5XFAD mice have mutations in the *APP* SweK670N/M671L, LonV717I, and FloI716V) and *PSEN1* (M146L and L286V) genes regulated by the Thy1 promoter.	DG: ↑Ki-67^+^, DCX^+^	Not given	Shin et al. (2018)
Shenzao jiannao oral liquid (SZJN)	Ginseng Radix et Rhizoma，Ziziphi Spinosae Semen， Celastrus Orbiculatus Thunb，Epimedii Folium，Rehmanniae Radix，Gastrodiae Rhizoma，Chrysanthemi Flos，Zingiberis Rhizoma，Glycyrrhizae Radix et Rhizoma.	Kunming mice (half-male and half-female); AD mouse model caused by a combination of A*β*42 and scopolamine	DG: ↑BrdU^+^, Nestin^+^ cortex and DG: ↑NeuN^+^	↑ The learning and memory abilities of Morris water maze test	Xiao et al. (2020)
		NSCs were obtained from hippocampal tissues of neonatal C57BL/6 mice； transfect NSCs with APP695swe and GFP genes	32 mg/ml of SZJN promote NSCs proliferation	Cell	Xiao et al. (2020)

Traditional Chinese medicine prescriptions could improve neurological diseases, some of which were found to be related to adult neurogenesis. At present, it has been reported that the major neurological diseases improved by TCMPs mainly include AD, stroke, and traumatic brain injury (TBI), and these diseases are all related to abnormalities of neurogenesis. From a functional perspective, promoting adult neurogenesis plays an important role in structural plasticity and network maintenance in AD (Mu and Gage, 2011). Currently, five types of TCMPs are being used in the treatment of AD; four of them improved the cognitive function in AD, and one reduced amyloid-*β* (Aβ) aggregation and Aβ-mediated pathology. Different TCMPs may improve the behavioral or pathological abnormalities of AD by acting at different stages of neurogenesis. Fuzhisan (Yang et al., 2011) and yokukansan (Azuma et al., 2018) acted at the proliferation level, Shenzao Jiannao oral liquid acted at the proliferation and maturation stages (Xiao et al., 2020), and herbal formula PM012 acted at the differentiation and maturation stages (Ye et al., 2016). Moreover, Jowiseungchungtang (Shin et al., 2018) inhibited Aβ-mediated pathology in an AD animal model (5XFAD) and restored adult neurogenesis in the proliferation and differentiation stages. Nine types of TCMPs have been shown to be effective in treating stroke, which is another common neurological disease. Seven of these TCMPs increased post-stroke brain function, and two of them improved both brain function and cognition after stroke. The above-mentioned nine TCMPs improved the restoration of brain function after stroke by promoting neurogenesis proliferation, differentiation, and maturation. Danggui Shaoyao San (Ren et al., 2015), Huatuo zaizao pill (Duan et al., 2017), and Sanhua decoction (Fu et al., 2020) acted at the differentiation stage. Tongxinluo (Chen L. et al., 2014; Chen et al., 2016) and Gualou Guizhi decoction (Han et al., 2018) all had an effect on the proliferation and differentiation levels. Huang-Lian-Jie-Du decoction (Zou et al., 2016) and Danggui jakyak San (Song et al., 2013) targeted the stages of proliferation, differentiation, and maturation of neurogenesis. Significantly, Buyang Huanwu decoction (Wang et al., 2011; Liu et al., 2013; Chen H. J. et al., 2015; Chen et al., 2020; Zhuge et al., 2020) and Ginseng Angelica shansheng pulvis (Liu et al., 2019) not only improved post-stroke brain function but also improved cognition, which may be related to the action of these two TCMPs on the proliferation, differentiation, and maturation stages of neurogenesis. In addition, after TBI, modified “Shengyu” decoction (Chen M. M. et al., 2015) improved neurological function, and MLC901 (Quintard et al., 2014) restored cognitive function, which may be related to the fact that these two TCMPs promoted the proliferation of the NCS (Figure 2).

**Figure 3:**
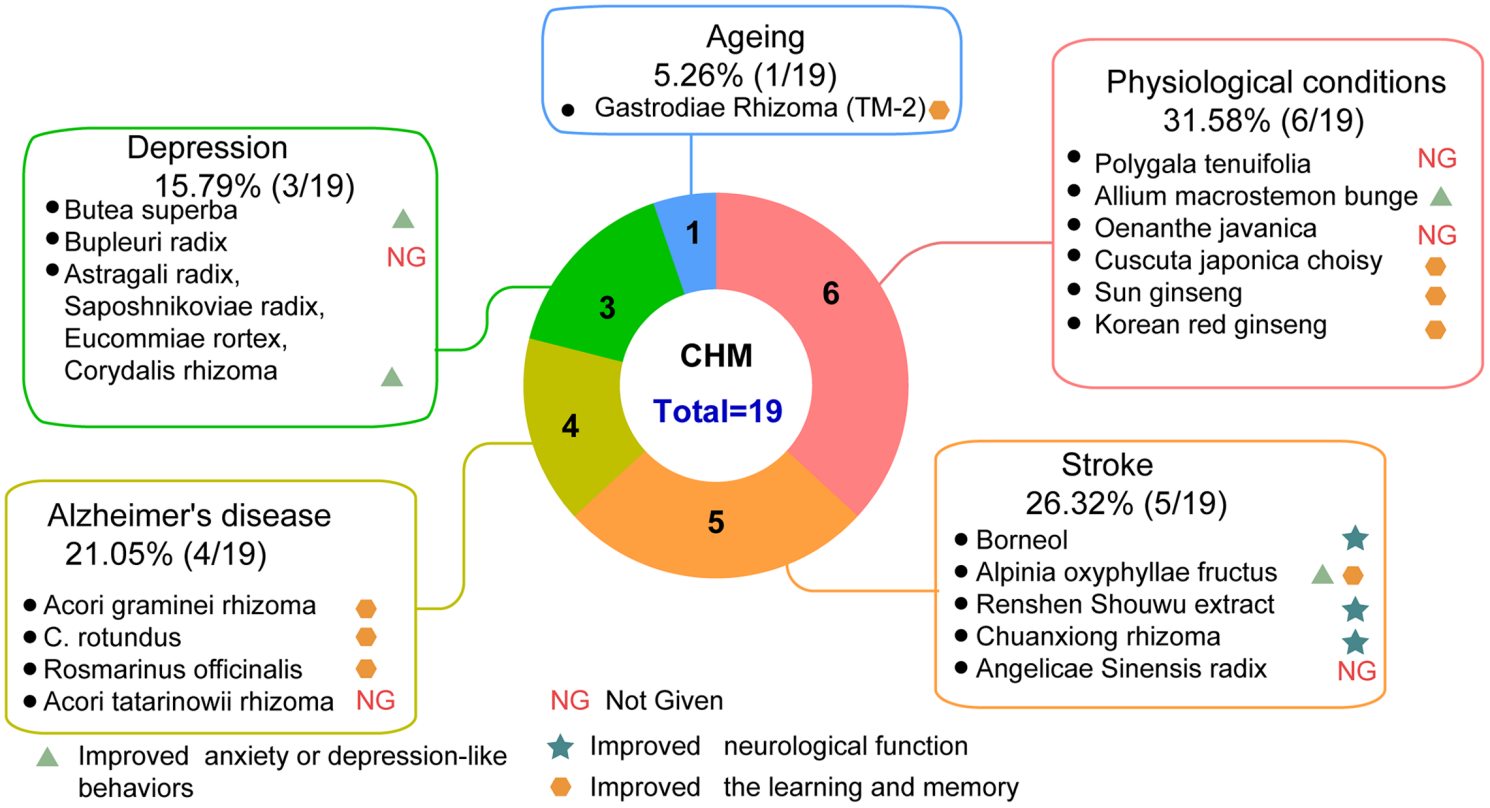
The effect of Chinese herbal medicine on adult neurogenesis and brain function, *n* = 19.

The most important psychiatric disorder improved by TCMPs is depression. Depression is associated with impairments in adult neurogenesis in the dentate gyrus, while the effects of antidepressants are mediated by increased neurogenesis. Increasing adult hippocampal neurogenesis could reduce anxiety and depression-like behaviors (Hill et al., 2015; Tunc-Ozcan et al., 2019). At present, a total of ten TCMPs alleviated the mental symptoms of depression, with nine of them significantly improving anxiety and depression-like mood after depression; one TCMP not only alleviated mood but also improved cognition. The aforementioned TCMPs that improve cognition and mood in depression may act on different stages of neurogenesis. Kami-shoyo-san (Park et al., 2007), Kososan (Ito et al., 2017), and Wuling capsules (Li et al., 2010) promoted the proliferation stage; Jiaweisinisan promoted the differentiation stage (Wang H. Z. et al., 2021); Kaixinsan promoted the maturation stage (Yan et al., 2016; Dong et al., 2020); Jie Yu Chu fan capsules (Ji et al., 2020), Xiaochaihutang (Zhang et al., 2015, 2016; Ma J. et al., 2017) and Zhengtian capsules (Yang et al., 2020) promoted the proliferation and differentiation stages; Chaihu-Shugan-San (Chen et al., 2018; Zhang et al., 2021) promoted the differentiation and maturation stages. In addition to reducing depressive symptoms, Ninjinyoeito (Murata et al., 2018) also improved cognitive performance, which may be connected to promoting the proliferation and differentiation stages of neurogenesis. Meanwhile, Kososan (Ito et al., 2017) improved mood, but it simply tended to advance the stage of proliferation. In addition, post-stroke depression (PSD) is a significant social and public health issue, and antidepressant preventive and curative treatments are worth investigating (Villa et al., 2018). TCMs not only ameliorated depression by affecting neurogenesis but also alleviated the symptoms of PSD by promoting the maturation of neurogenesis. Both Yi-nao-jie-yu (Tian et al., 2018) and Jieyu Anshen granules (Du et al., 2020) relieved the mood after PSD, restored brain function, and improved cognitive function; this may be related to the fact that these two TCMPs promoted NSC maturation.

In addition, under physiological conditions, Kami-ondam-tang (Hong et al., 2011) is good for cognition, and Xiaochaihutang (Zhang et al., 2015) is beneficial for emotion, which may be related to the fact that these TCMPs are able to promote the differentiation of neural stem cells.

The application of each of the above 29 types of TCMPs is based on the theory of TCM and has been consistently enhanced through the process of practice. As a result, neurogenesis has been improved in a variety of situations. It is evident that each TCMP contains several different herbs, but identifying which ones are the most important may be difficult to explain. Future research will focus on those factors that support adult neurogenesis and have either antagonistic or synergistic effects.

### 3.2. The effects of Chinese herbal medicine on adult neurogenesis

According to the “jun-chen-zuo-shi” principle of TCM, each CHM in a TCMP is essential and has a specific function (Zhang et al., 2014). The advancement of modern pharmacology has made it easier to further study the active components in TCM that promote adult neurogenesis. Therefore, the effects of CHMs on adult neurogenesis have been widely studied. Table 2 and Figure 3 summarizes the impact of CHMs on adult neurogenesis under different pathological and physiological situations.

**Table 2:** Effects of CHMs on neurological conditions and adult neurogenesis.

Herbs	Extraction method	Animals/cell types	Outcome measurement	Aspects of behaviors/function	References
Polygalae Radix	EtOH extract	Adult male Sprague–Dawley rats	CA1:↑ BrdU^+^. ↑ Nestin^+^/BrdU^+^, Tuj1^+^/BrdU^+^	Not GIVEN	Park et al. (2008)
		HiB5(rat neuronal precursor cells)	↑Promoted the neurite outgrowth	Cell	Park et al. (2008)
Allium macrostemon Bunge (AM-W)	Water extract	Male ICR mice	CA1:100 mg/kg: ↑ DCX^+^, NeuN^+^/BrdU^+^ 200 mg/kg: ↑ BrdU^+^,DCX^+^,NeuN^+^/BrdU^+^	↓The immobility duration of the forced swimming test ↓the immobility duration of the tail suspension test	Lee et al. (2010)
Sun ginseng	EtOH extract	Male ICR mice	DG: 20 mg/kg: ↑ BrdU^+^,DCX^+^	↑ The step-through latency	Lee et al. (2013)
*Oenanthe javanica*	EtOH extract	male Wistar rats	DG: ↑ DCX^+^, Ki-67^+^	Not GIVEN	Chen B. H. et al. (2015)
Acori tatarinowii Rhizoma	EtOH extract	C57BL/6 mice	DG:↑BrdU^+^、Ki67^+^ ↑Tbr2^+^/BrdU^+^ ↑DCX+/Ki67+, DCX+/Ki67 ↑BrdU^+^/NeuN^+^	Not GIVEN	Mao et al. (2015)
		NPCs from hippocampal of C57BL/6 mice	↑EDU^+^	Cell	Mao et al. (2015)
Cuscutae Semen	Water extract	Male ICR mice	DG: 10 mg/kg/day: ↑ BrdU^+^/ NeuN^+^ 50 mg/kg/day:↑Ki-67^+^, DCX^+^, BrdU^+^/ NeuN^+^ 100 mg/kg/day:↑Ki-67^+^, DCX^+^, BrdU^+^/DCX^+^, BrdU^+^/ NeuN^+^	↑ Time exploring the novel object	Moon et al. (2016)
Korean red ginseng		C57BL/6 mice	DG: ↑BrdU^+^, BrdU^+^/DCX^+^	↑ The learning and memory abilities of Morris water maze test	Ryu et al. (2020)
Astragali Radix, Saposhnikoviae Radix, Eucommiae Cortex, Corydalis Rhizoma	Water extract	The mouse NSC line (mNSC 9,601)	↑ cell proliferation (MTS assay)	Cell	Sun et al. (2016)
		BALB/c mice; chronic mild stress (CMS) was used in mice for 14 days to establish a depression-like mouse model.	DG: ↑BrdU^+^	↑ The body weight gain ↓ the duration of immobility in the FST	Sun et al. (2016)
Bupleuri Radix	Water extract	Oxidative stress induced by serum deprivation in SH-SY5Y cells	↑ BrdU^+^	Cell	Seo et al. (2013)
Butea superba (BS)	EtOH extract	72 male ddY mice were obtained at the age of 7 weeks old, The UCMS group received various unpredictable stressful stimuli for 7 weeks	DG↑ DCX^+^	↑The sucrose intake ↓the immobility times (tail suspension test)	Mizuki et al. (2014)
Acori graminei Rhizoma (AGR)	Water extract, volatile oil fraction, or defatted decoction fraction of AGR	Alzheimer disease-like symptoms induced by Amyloid Beta (Aß) 1–42 intra-hippocampal injection for 7 days	DG↑ DCX^+^, Nestin^+^	↑Spatial memory (Morris water maze)	Ma et al. (2015)
Rhizoma Acori tatarinowii		aged C57BL/6 mice (age at 18–23 months);8-month-old middle-aged APP/PS1 mice	DG aged mice: ↑ BrdU^+^,BrdU^+^/NeuN^+^ APP/PS1:↑BrdU^+^,Ki67^+^,BrdU^+^/NeuN^+^	Not given	Mao et al. (2015)
*C. rotundus*	EtOH extract	Wistar rats received 5 ug/ul Aβ1-42 into CA1 bilaterally for AD	DG: ↑NeuN^+^	↑Spatial memory (Morris water maze test)	Shakerin et al. (2020)
Gastrodiae Rhizoma (TM-2)	EtOH extract	C57BL/6 mice, The D-gal groups were subcutaneously injected with 200 mg/kg D-gal daily for 8 weeks to establish the aging model.	DG:↑BrdU^+^,DCX^+^	↑Spatial memory (Morris water maze test); Burrowing and nesting behaviors	Hsu et al. (2021)
Rosmarinus officinalis	EtOH extract	BALB/c male mice; Aβ1-42 peptide (dilution 1 μg per μl) was injected into the CA1 area of the hippocampus for AD	Hippocampus(mRNA):↑Ki67^+^;DCX^+^; NeuN^+^	↑Spatial memory (Morris water maze test) and object recognition memory (NOR test) exhibit anti-anxiety effects (the elevated plus maze test)	Mirza et al. (2021)
Borneol	Borneol was dissolved in 5% Tween 80 and given to mice by gavage	C57BL/6 mice, with Focal Cerebral Ischemia–Reperfusion Model	Infarct zone:↑NeuN^+^ ↓GFAP^+^	↑ Neurological score and global score	Zhang X. et al. (2017) and Zhang X. G. et al. (2017)
Alpiniae Oxyphyllae Fructus	EtOH extract	C17.2 cells exposed to 4-h OGD plus 20-h reoxygenation	*p*-coumaric acid:↑BrdU^+^/Ki67^+^ ↑BrdU^+^/SOX2 ^+^	Cell	He et al. (2020)
		Sprague–Dawley (S.D.) rats were subjected to MCAO to induce cerebral ischemia animal model	DG and SVZ: *p*-coumaric acid:↑BrdU^+^/Ki67^+^, BrdU^+^/DCX^+^, BrdU^+^/NeuN ^+^in the	↑ Body Weight; spatial learning/memory (Morris water maze test) and recognition capacity (NOR test), ↓anxiety (open-field test)	He et al. (2020)
Renshen Shouwu extract	EtOH extract	Sprague–Dawley rats with Middle cerebral artery occlusion (MCAO) surgery	Penumbra: ↑NeuN^+^/BrdU^+^	↑Neurological function (the Zea Longa’ method)	Li et al. (2020)
Chuanxiong Rhizoma	EtOH extract	Wistar rats was induction of microsphere-induced cerebral embolism (ME) FOR ischemia	DG: ↑DCX^+^	↑Neurological score after operation at 1, and 3 days, respectively	Wang et al. (2020)
Angelicae Sinensis Radix	Water extract	Sprague–Dawley rats; global cerebral ischemia (GCI) was induced in the rats using the 4-vessel occlusion (4-VO) method	ASD-0.5 g, and ASD-1 g: SGZ: ↑BrdU^+^ and BrdU^+^/NeuN^+^ SGZ: ↑Ki67^+^ and Ki67^+^/nestin^+^ CA1: ↑MAP-2^+^/NeuN^+^	Not GIVEN	Cheng et al. (2021)

The main neurological diseases that CHMs could improve are AD and stroke, and this improvement in neurological symptoms may be related to neurogenesis. Four CHMs promoted neurogenesis in AD animal models, and three of them improved the cognition of AD animals, but they had different effects on neurogenesis. Acori graminei rhizoma mainly acted on proliferation and differentiation (Ma et al., 2015), Rosmarinus officinalis mainly acted on differentiation (Mirza et al., 2021), and Cyperus rotundus mainly acted on maturation (Shakerin et al., 2020). In addition, Acori tatarinowii rhizoma (Mao et al., 2015) promoted the proliferation and maturation of neurogenesis in AD animal models, but its effect on cognition has not been shown. Five CHMs have improved brain function after an ischemic stroke. The restored brain function after stroke may be related to chuanxiong rhizome-stimulated differentiation (Wang et al., 2020), Borneol (Zhang X. G. et al., 2017), and Renshen Shouwu extract stimulated maturation (Li et al., 2020). Meanwhile, Alpiniae oxyphyllae fructus improved cognition and mood after stroke, which may be related to its promotion of cell proliferation, differentiation, and maturation (He et al., 2020).

Three reports indicate that CHMs improved the neurogenesis of depression Butea superba (Mizuki et al., 2014); Astragali radix, Saposhnikoviae radix, Eucommiae cortex, and Corydalis rhizoma (Sun et al., 2016) all have the potential to lessen depression, and one of the possible mechanisms is the promotion of the proliferation of neurogenesis. In addition, Bupleuri radix is a key component in a number of oriental herbal medicines used to treat stress and other psychiatric illnesses, and these seem to have proliferative effects (Seo et al., 2013).

Six CHMs promote neurogenesis under physiological conditions, and three of these improved learning and memory, with one CHM alleviating emotion and two others promoting neurogenesis (however, their effects on cognition and emotion were not demonstrated). The following three CHMs have been shown to improve learning and memory. Their potential mechanisms may involve sun ginseng, which promotes NSC proliferation and survival (Lee et al., 2013), Korean red ginseng, which promotes NSC differentiation (Ryu et al., 2020), and Cuscuta japonica Choisy, which promotes NSC proliferation, differentiation, and maturation (Moon et al., 2016). Allium macrostemon Bunge (Lee et al., 2010) was beneficial to antidepressant-like activity and promoted the proliferation, differentiation, and maturation of neurogenesis. In addition, Oenanthe javanica encouraged neurogenesis proliferation and differentiation (Chen B. H. et al., 2015), and the root of Polygala tenuifolia encouraged neurogenesis proliferation and maturation (Park et al., 2008), although their effects on cognition and emotion under physiological situations were not demonstrated. In Asia, certain CHMs, such as Korean red ginseng, are used as both medicine and food.

### 3.3. The effects of bioactive components on adult neurogenesis

**Figure 4:**
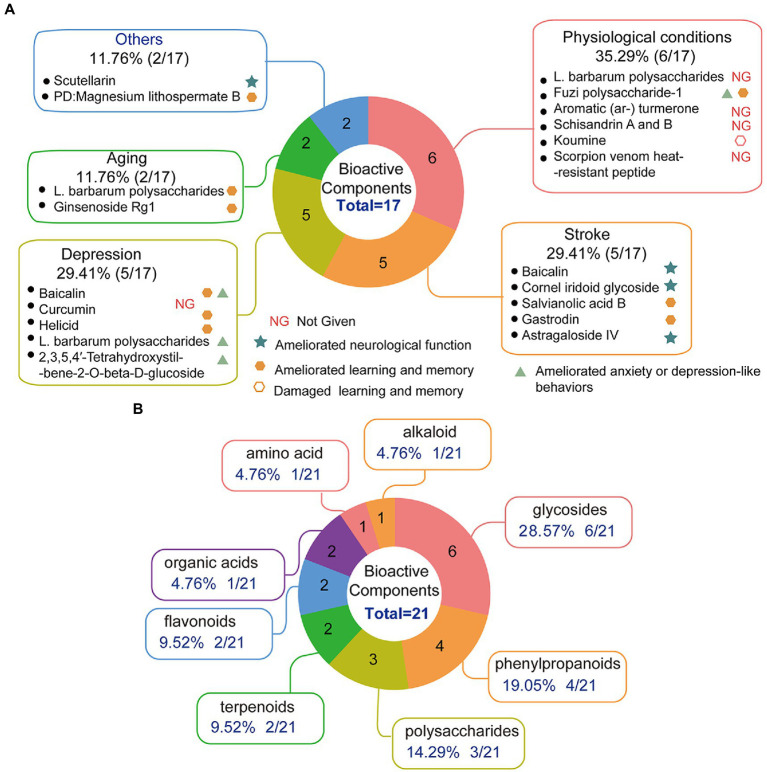
The effect of bioactive components on adult neurogenesis. **(A)** Pie chart of bioactive components improving adult neurogenesis and brain function, *n* = 17, *L. barbarum* polysaccharides improves adult neurogenesis in aging, depression, and physiological conditions, so it was counted in three cases for three situations; baicalin improves adult neurogenesis in depression and stroke conditions, so it was counted in two cases for two situations. **(B)** Pie chart of bioactive components according to the chemical composition that promotes adult neurogenesis (animal) or contributes to the proliferation and differentiation of NSCs, *n* = 21.

The bioactive components were extracted from CHMs due to their structural diversity and biological activities, which make them important sources of clinical drugs. Although there are fewer components in CHMs than in TCMPs, it is still very difficult to determine which components are effective. Therefore, the separation and extraction of bioactive components from CHMs for research provide a more stable outcome and may be conducive to the study of pharmacological mechanisms. The destiny of NSCs may be influenced by bioactive components (Zhang Z. et al., 2018), and bioactive components’ support of adult neurogenesis has attracted extensive attention. Table 3 and Figure 4A show the 17 kinds of bioactive components that were reported to improve adult neurogenesis in connection to physiological or pathological conditions. The effects of bioactive components on adult neurogenesis have been studied at the cellular and animal levels (Table 3).

**Table 3:** The bioactive components of neurological conditions and adult neurogenesis.

Bioactive components	Source	Animals/cell types	Outcome measurement	Aspects of behaviors/function	References
*In vivo*
Baicalin	Scutellariae Radix	KunMing mice; MCAO model	Baicalin performed well in regulating proteins in energy metabolism but had a relatively weak effect in the regulation of proteins in neurogenesis and apoptosis	↑Nissl’s bodies	Zhang et al. (2009)
Cornel iridoid glycoside (CIG)	Corni Fructus	Sprague–Dawley rats; The middle cerebral artery occlusion was induced for MCAO	CIG (60 and 180 mg/kg/day):↑ BrdU^+^, Nestin^+^ in the ischemic ipsilateral SVZ 14–28 days after MACO,	↑ Neurological function (Modified neurological severity score)	Yao et al. (2009)
Salvianolic acid B	Salviae Miltiorrhizae Radix et Rhizoma	Wistar rats were subjected to transient forebrain ischemia	DG: 50 mg/kg Sal B ↑BrdU^+^	↑ The learning and memory ability (Morris water-maze)	Zhuang et al. (2012)
Gastrodin	Gastrodiae Rhizoma	C57BL/6 J mice with *cerebral ischemia*	DG: Day15: ↑BrdU^+^, DCX^+^ in the dentate gyrus. Day29:↑BrdU^+^/NeuN^+^ cells,	↑Spatial memory (Morris water-maze)	Xiao et al. (2021)
Astragaloside IV	Astragali Radix	Sprague–Dawley rats; middle *cerebral artery occlusion/reperfusion model*	Peri-ischemic regions: ↑BrdU^+^/NeuN^+^ and BrdU^+^/GFAP^+^	↑ *Neurological function recovery* (modified neurological severity score) ↓infarct volume (toluidine blue solution)	Li et al. (2021)
		Sprague–Dawley (SD) rats with Cerebral Ischemia–Reperfusion Model	SVZ and DG: ↑BrdU^+^/SOX2^+^, BrdU^+^/DCX^+^, BrdU^+^/NeuN^+^-positive staining cells ↑BrdU^+^/GFAP^+^	↑Spatial memory (Morris water-maze test), and Motor Function (the rotarod test) Recovery	Chen et al. (2019)
Baicalin	Scutellariae Radix	Sprague–Dawley rats；corticosterone groups at a dose of 40 mg/kg daily for 14 days to induce stress.	DG: ↑DCX^+^	Not given	Jiang et al. (2013)
		C57BL/6 N mice were exposed to chronic CORT treatment (70 μg/ml/day, 30 days). APPL2 Tg mice (male, 8 weeks)	↑BrdU^+^、BrdU^+^/NeuN^+^in DG region; ↑BrdU^+^/NeuN^+^ cells in the GCL areas;6.7 mg/kg/day:↑BrdU^+^/DCX^+^, density of neuronal progenitors at both dorsal and ventral SVZ regions	↓The CORT-Induced Depressive- and Anxiety-Like Behaviors (tests including splash, tail suspension test, forced swim test, open field test, and novelty suppressed feeding)	Gao et al. (2018)
		Male ICR mice CUMS for 6 weeks	DG: ↑DCX^+^, BrdU^+^/NeuN^+^ the SGZ	↑ Sucrose consumption, the number of crossings in open filed test and ↓the immobility time in tail suspension test	Zhang et al. (2019)
Curcumin	Xiaoyao-san	Sprague–Dawley (SD) rats, stress was administered once per day over a period of 20 days	DG:(at 10 and 20 mg/kg): ↑BrdU^+^	Not given	Xu et al. (2007)
2,3,5,4′-Tetrahydroxystilbene-2-O-beta-D-glucoside (THSG)	Polygoni Multiflori Caulis	C57BL/6 mice chronic restraint stress for 28 days	Hippocampus: (40 mg/ml): ↑DCX^+^	Depressive-like behaviors in CRS mice as measured by the tail suspension test, forced swimming test, and open-field test.	Jiang et al. (2018)
Helicid	Helicia nilagirica	Sprague–Dawley (SD) rats; Chronic Unpredictable Mild Stress for 12 weeks	CA1 and DG: ↑BrdU^+^ in the	↑body weight; sucrose consumption, distance and number of crossings in the open-field test (OFT), ↓the immobility times in the forced swimming test (FST) and improved spatial memory in the Morris water maze (MWM);	Li et al. (2019)
Fuzi polysaccharide-1	Aconiti Lateralis Radix Praeparata	Male C57BL/6 J mice	DG: A single injection of FPS (10–400 mg/kg):↑BrdU^+^ in the DG; FPS (100 mg/kg,7 Days):↑NeuN^+^/BrdU^+^、the proportion of NeuN^+^/BrdU^+^ cells to the total number of BrdU^+^	↓ Immobility in the forced swim test, and latency in the novelty suppressed-feeding test.	Yan et al. (2010)
Magnesium lithospermate B	*Salviae Miltiorrhizae Radix et Rhizoma*	PD rat model	↑Ki67^+^, Thy1^+^, in DG region ↑MAP2^+^ and PSD95 in hippocampal region	↑Spatial memory (Morris water maze)	Zhang J. H. et al. (2018), Zhang R. et al. (2018), Zhang Z. et al. (2018)
*L. barbarum* polysaccharides (LBP)	Lycii Fructus	Sprague–Dawley rats; Scopolamine-Treated Rats pumps 440 mg/ml of SCO solution were subcutaneously embedded in abdominal wall SCO release (0.25 ml/h) was maintained for 28 days	DG: ↑Ki67^+^, DCX^+^	↑ Time exploring the novel object or location in the recognition tasks ↓ escape latency in the water maze.	Chen W. et al. (2014)
		Sprague–Dawley rats received daily i.p. injection with 40 mg/kg dextromethorphan for 14 days	DG: ↑DCX^+^, ↑DCX^+^/BrdU^+^	Alleviated DXM-induced depression-like (forced swim test) and social anxiety-like behaviors (social interaction test)	Po et al. (2017)
		Sprague–Dawley rats	↑Ki-67^+^	Not given	Wang et al. (2015)
Scutellarin	*Erigeron breviscapus* Hand-Mazz	C57BL/6 mice were exposed to cuprizone (8 mg/day) *via* food intake (0.2% cuprizone in standard rodent chow) for 6 weeks	SVZ: ↑Sox2^+^, Nestin^+^	↓ The motor deficit (rotarod test)	Wang et al. (2016)
Aromatic (ar-) turmerone	Curcumae Longae Rhizoma	Spontaneously breathing male Wistar rats； single intracerebroventricular injection of 3 mg ar-turmerone at a concentration of 1 mg/μl.	SVZ: ↑DCX^+^ in	Not given	Hucklenbroich et al. (2014)
Scorpion venom heat-resistant peptide	Scorpio	C57BL/6 male mic	SGZ and OB: ↑BrdU^+^, BrdU^+^/NeuN^+^,PSA-NCAM^+^ SGZ and SVZ: ↑GFAP^+^/ Nestin^+^ radial glia-like precursors	Not given	Wang et al. (2014)
Schisandrin A and B	Schisandrae Chinensis Fructus	Kunming White mice	DG: Sch A:↑GFAP^+^, NeuN^+^ Sch B: ↑PHH3^+^, GFAP^+^, NeuN^+^	Not given	Cai et al. (2020)
Koumine	Gelsemium elegans Benth	Both male and female c57BL/6 J mice	SGZ: ↓DCX^+^, BrdU^+^, BrdU^+^/DCX^+^ in the	Prenatal KM:↓cognitive and memory (Morris water maze, Y-maze test, and novel object recognition test), long-term potentiation Prenatal KM offspring: ↑Anxiety-like behavior (the open field test and elevated plus maze test)	Yang et al. (2021)
*In vitro*					
Salvianolic acid B	Salviae Miltiorrhizae Radix et Rhizoma	Primary neurospheres were from the cerebral cortex of 13.5-day-embryonic Wistar rats	Promoting NSPCs proliferation. ↑Nestin and Notch-1	Cell	Zhuang et al. (2012)
Magnesium lithospermate B	Salviae Miltiorrhizae Radix et Rhizoma	NSCs were from the hippocampal of newborn mice. Wide type newborn C57BL/6 mice (P1 age)	Increasing effect reached the maximum around the concentration of 10 μg/ml, and maintained its effect on proliferation of NSCs at 50 and 100 μg/ml	Cell	Zhang J. H. et al. (2018), Zhang R. et al. (2018), Zhang Z. et al. (2018)
Angelica polysaccharide	Angelicae Sinensis Radix	NSCs	ASP increased the cell proliferation, and proliferation viability of ASP treated NSCs was dose-dependent (0–160 ug/mL).	Cell	Cheng et al. (2019)
Astragaloside IV	Astragali Radix	Neural progenitor cell line C17.2 cells (1 × 104 cells/ml).	↑ BrdU^+^, the diameters of neurosphere, and cell viability	Cell	Chen et al. (2019)
Saikosaponins-d	Bupleuri Radix	Primary NPCs were isolated from the hippocampus of newborn C57BL/6 J mice	Dose dependent decrease in cell viability in NPCs. NPCs were incubated with SSd (2, 4 μM) for 24 h:↓Edu^+^, Ki67^+^	Cell	Qin et al. (2019)
Tetramethylpyrazine	Chuanxiong Rhizoma	SH-SY5Y human neuroblastoma cells	Western blot analysis showed that MAP2 and tau started to increase from 5 days and 3 days, respectively, after treatment with TMP	Cell	Yan et al. (2014)
Musk ketone	Musk	Brain tissues from neonatal rats were aseptically obtained for NSC Establishment of oxygen–glucose deprivation (OGD) cell model *in vitro*	Treated with 0.9 lM or 1.8 lM musk ketone: ↑BrdU^+^/Tju-1^+^ and BrdU^+^/vimentin^+^ cells	Cell	Zhou et al. (2020)
Epimedium flavonoids	Epimedii Folium	Hippocampi from neonatal 1-day rats were isolated and mechanically triturated	10, 50 mg/ml: ↑axons’ lengths 100 mg/ml: ↑average migration distances、axons’ lengths 200 mg/ml:↑neurospheres	Cell	Yao et al. (2010)
Aromatic (ar-) turmerone	Curcumae longae Rhizoma	NSCs were cultured from fetal rat cortex at embryonic day 14.5	1.56 μg/ml: ↑BrdU^+^ 3.125 μg/ml:↑ cell number, BrdU^+^ 6.25 μg/ml:↑ cell numbers, BrdU^+^, Ki67^+^, SOX2^+^	Cell	Hucklenbroich et al. (2014)

At present, *in vivo* experiments have shown that bioactive components are being used to treat neurologic diseases such as stroke, PD, multiple sclerosis, and aging. Five bioactive components improved symptoms after stroke, promoting neurogenesis. Cornel iridoid glycoside (Yao et al., 2009) restored brain function, which may be related to NSC proliferation. Learning and memory after stroke were improved by salvianolic acid B (Zhuang et al., 2012) and Gastrodin (Xiao et al., 2021). This improvement may have been caused by the stimulation of neurogenesis. Astragaloside IV (Chen et al., 2019; Li et al., 2021) not only restored brain function after stroke but also improved cognition, which may have the potential to encourage NSC proliferation, differentiation, and maturation. Baicalin also performed well in regulating proteins in energy metabolism, but had a relatively weak effect in the regulation of proteins in neurogenesis and apoptosis, according to results from proteomics to explore the various protein expression modes in mouse brains after stroke (Zhang et al., 2009). Magnesium lithospermate B (Zhang Z. et al., 2018) improved the cognitive function of PD animal models, which may be connected to NSC growth promotion. Scutellarin (Wang et al., 2016) alleviated behavioral deficits in a mouse model of multiple sclerosis, possibly by inhibiting NSC apoptosis and promoting NSC differentiation into myelin-producing oligodendrocytes. *Lycium barbarum* polysaccharides (LBP) (Chen W. et al., 2014) prevented cognitive and memory deficits, in addition to decreasing cell proliferation and neuroblast differentiation, in scopolamine-treated rats. Ginsenoside Rg1 prevented cognitive impairment in a rat model of aging (Zhu et al., 2014). This may be related to its ability to protect NSCs/NPCs and promote differentiation. Depression is the primary psychiatric illness alleviated by the bioactive components. Five bioactive components improved the symptoms of depression, with four of them relieving depression-like mood. This may be related to the fact that these four bioactive components can affect the neurogenesis of depression in model mice. Curcumin promoted proliferation (Xu et al., 2007), 2,3,5,4 ‘-Tetrahydroxystilbene-2-*O*-beta-d-glucoside (Jiang et al., 2018), and LBP (Po et al., 2017) promoted differentiation, and baicalin promoted proliferation, differentiation, and maturation (Jiang et al., 2013; Gao et al., 2018; Zhang et al., 2019). Meanwhile, Helicid (Li et al., 2019) not only relieved post-depression mood but also improved cognition, which may be associated with boosting NSC proliferation. Also, under physiological conditions, bioactive components promoted neurogenesis. For example, Fuzi polysaccharide-1 (Yan et al., 2010) improved mood, which may have been connected to its support of proliferation and maturation. The effect of LBP promoted proliferation (Wang et al., 2015), aromatic Turmerone (Hucklenbroich et al., 2014), and schisandrin A and B (Cai et al., 2020) promoted differentiation, scorpion venom heat resistant peptide promoted proliferation and differentiation (Wang et al., 2014), but the effects above five bioactive components on the behaviors of mice have not been reported.

*In vitro*, bioactive components mainly promoted the proliferation and differentiation of NSCs. Magnesium lithospermate B (Zhang Z. et al., 2018), angelica polysaccharide (Cheng et al., 2019), and astragaloside IV (Chen et al., 2019) all promoted the proliferation of stem cells; tetramethylpyrazine (Yan et al., 2014) and musk ketone promoted differentiation, while salvianolic acid B (Zhuang et al., 2012) and aromatic turmerone (Hucklenbroich et al., 2014) regulated both proliferation and differentiation. In addition, Epimedium flavonoids promoted axon growth, which is essential for stem cell maturation (Yao et al., 2010). Unfortunately, when pregnant rats were exposed to koumine (Zhou et al., 2020), which was isolated from Gelsemium elegans Benth, the offspring of both male and female c57BL/6 J mice showed a marked reduction in neurogenesis in the hippocampal DG. In addition, the offspring presented cognitive deficits and increased anxiety-like behavior (Yang et al., 2021). Similarly, Saikosaponin-d replicated cell viability and reduced cell growth (Qin et al., 2019).

The structural classification of the aforementioned bioactive components can be seen in Figure 4B. The indicated bioactive components are mainly concentrated in saponin (28.57%), phenylpropanoid (19.05%), and polysaccharide (14.29%), in addition to terpenoids (9.52%), organic acids (9.52%), amino acids (4.76%), alkaloids (4.76%), flavonoids (4.76%), and bioactive substances (4.76%).

**Figure 5:**
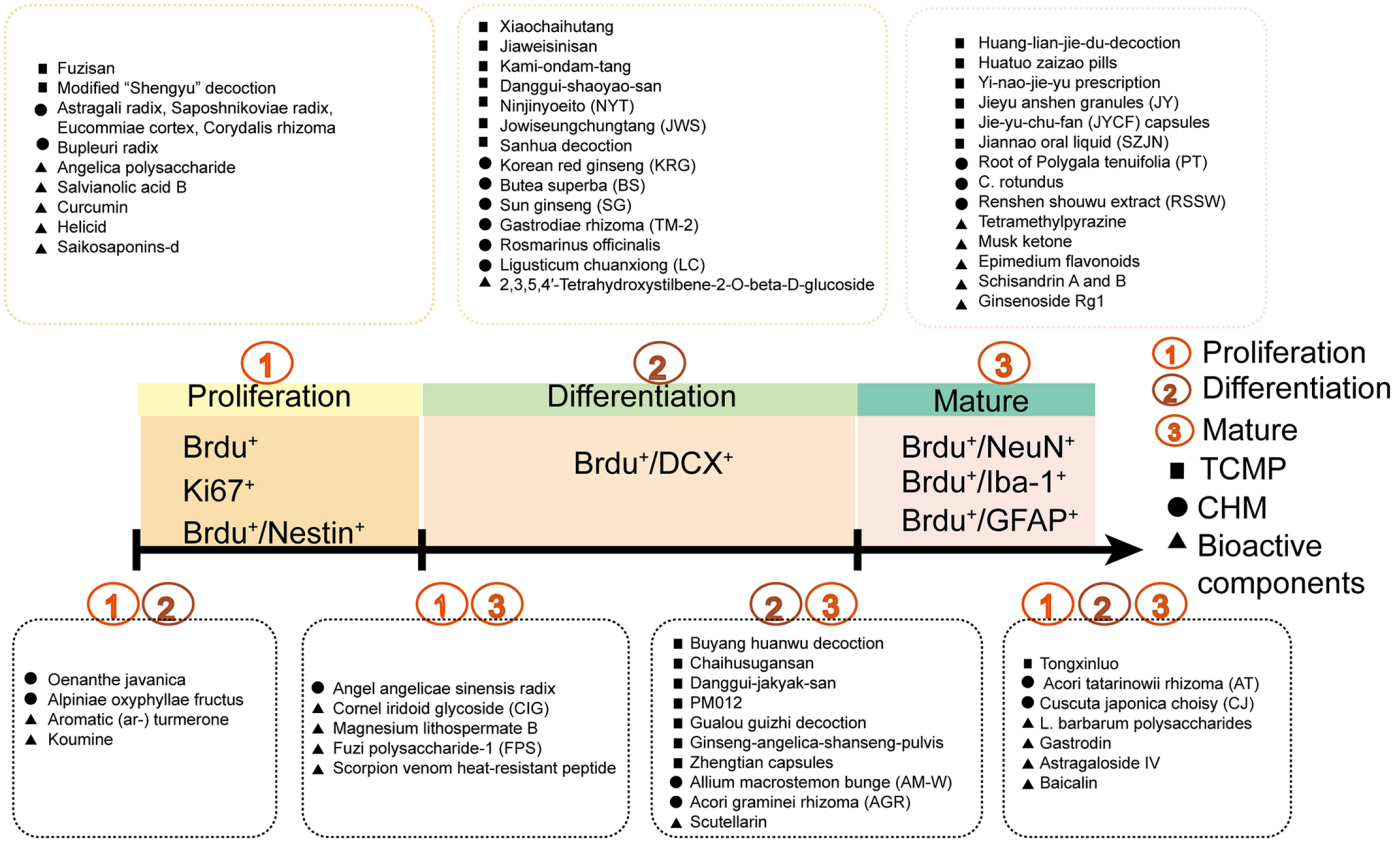
The influence of TCMPs, CHMs, and bioactive components on adult neurogenesis at different stages.

As people pay more attention to the role of neurogenesis in diseases, how to improve diseases through drugs that affect neurogenesis has become a hot subject in neuroscience in recent years. TCM has outstanding clinical efficacy in the treatment of neurogenesis-related diseases and is an important source of drugs that affect neurogenesis. TCMPs have a large amount of clinical practice data, such as Xiaochaihutang (Chen et al., 2009) and Buyang Huanwu decoction (Lee et al., 2020). Some herbs, such as medlar, ginseng, and licorice can be used as both medicine and food. Surprisingly, 9 of the 21 CHMs (47.6%) were shown to enhance adult neurogenesis under physiological conditions. When it comes to the different stages of neurogenesis, TCM may regulate more than just the one biological process of adult neurogenesis mentioned above. Figure 5 shows how TCM regulates and plays a vital role in the multi-stage process of adult neurogenesis.

**Figure 6:**
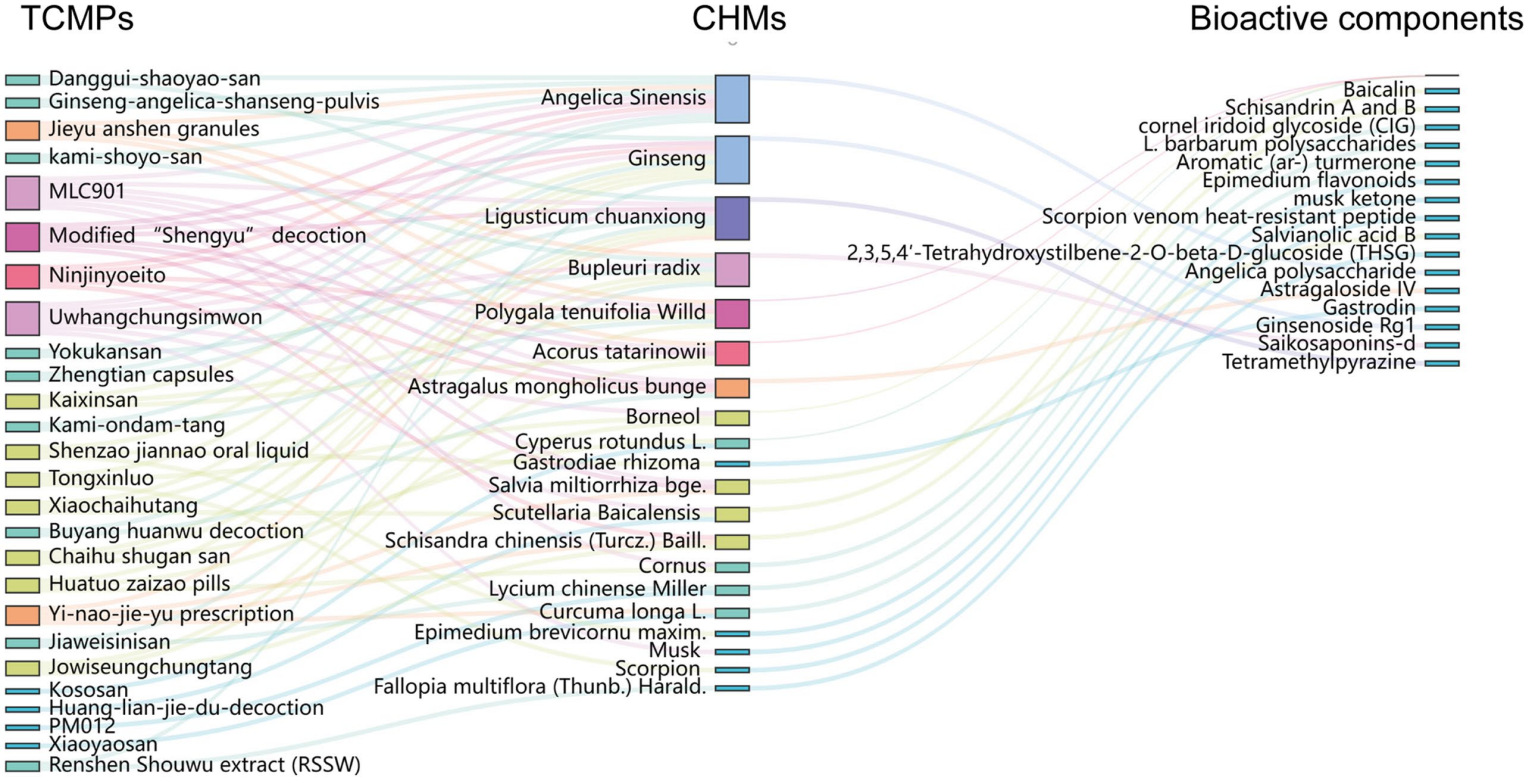
TCMPs and CHMs are commonly used to enhance adult neurogenesis and a network of its bioactive components.

As shown in Figure 6, there are currently 19 CHMs that are almost present in 26 TCMPs (89.66% of the total number of TCMPs), and further analysis found that 20 CHMs contain 17 types of bioactive compounds (80.95% of the total number of bioactive compounds), which have a high potential for use before clinical application, such as baicalin, which was isolated from the root of Scutellaria baicalensis and has a great neuroprotective effect. More importantly, baicalin has shown highly promising results in two clinical trials (chiCTR180016727 and ChiCTR180016727) on mental health. If we can fully explore the mechanism of its influence on neurogenesis, its clinical application will advance even further.

## 4. Mechanism of TCM on adult neurogenesis

### 4.1. Increase of neurotrophic factor

**Figure 7:**
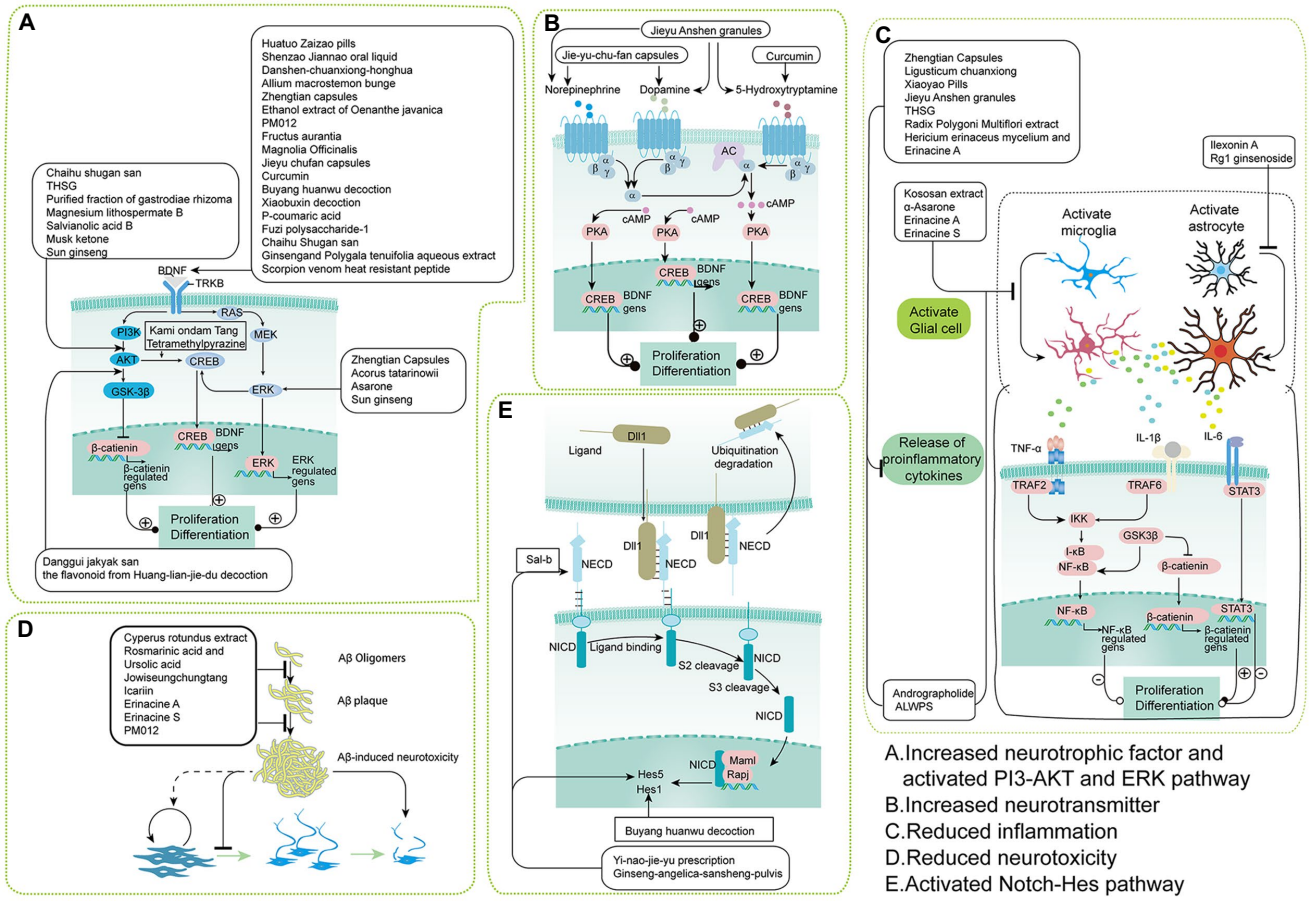
Mechanisms of TCMPs, CHMs, and bioactive components on adult neurogenesis. **(A)** TCM could increase neurotrophic factors or activate the PI3-AKT and ERK pathways for adult neurogenesis. **(B)** TCM could increase neurotransmitters for adult neurogenesis. **(C)** TCMs could reduce inflammation for adult neurogenesis. **(D)** TCM could reduce neurotoxicity for adult neurogenesis. **(E)** TCM could activate the Notch-Hes pathway for adult neurogenesis.

Neurotrophic factors play a central role in NSC proliferation, migration, and differentiation. Their existence is crucial for maintaining neuronal function, structural integrity, and adult neurogenesis throughout life. Many TCMs (Figure 7A) show the ability to promote the secretion of neurotrophic factors, thereby enhancing hippocampal adult neurogenesis (Zhang et al., 2014).

There are many TCMs that promote adult neurogenesis while simultaneously regulating BDNF, including Huatuo Zaizao pill (Duan et al., 2017), Shenzao jiannao oral liquid (Xiao et al., 2020), Danshen-Chuanxiong-Honghua (Zhang X. et al., 2017), and Allium macrostemon Bunge (Lee et al., 2010): these not only promote the proliferation of NSCs, but also enhance BDNF expression. Both Zhengtian capsules (Yang et al., 2020) and Oenanthe javanica ethanol extract (Chen B. H. et al., 2015) promote the proliferation and differentiation of hippocampal NSCs, while BDNF expression is also increased. PM012 promotes BDNF expression and NSC differentiation and maturation (Ye et al., 2016). In addition to the aqueous extract of gardenia, Fructus aurantii, and Magnolia officinalis also increase BDNF expression in the hippocampus of rats with chronic unpredictable mild stress and affect NSC differentiation and maturation (Xing et al., 2015). As a result, BDNF may be the target of traditional Chinese medicine to regulate neurogenesis. Thus, some researchers believe that the beneficial effect of Jieyu Chufan capsules (Ji et al., 2020) and curcumin (Xu et al., 2007) on depressed mice involves enhancing adult neurogenesis by boosting BDNF expression (Ji et al., 2020). Buyang Huanwu decoction promotes recovery from cerebral ischemia, and its mechanism may be related to the increased expression of VEGF and BDNF proteins for the differentiation and maturation of NSCs (Zhuge et al., 2020). PMC-12 (Park et al., 2016) and Xiaobuxin decoction (An et al., 2008) have beneficial effects on maturation through an increase in BDNF and p-CREB expression (An et al., 2008). Further research confirms that P-coumaric acid’s effects on BDNF/TrkB/Akt activation and NSC proliferation are eliminated when coupled with the BDNF/TrkB-specific inhibitor ANA12 (He et al., 2020). Meanwhile, K252a is an antagonist of Trk, an upstream molecule of BDNF signal transduction. TrkB inhibition blocks the transmission of the BDNF signal pathway. Although Fuzi polysaccharide-1 (Yan et al., 2010) promotes proliferation Chaihu Shugan San improves differentiation (Chen et al., 2018), and Ginseng and Polygala tenuifolia aqueous extracts (Jiang et al., 2021) enhance NSC differentiation and maturation, K252a may disrupt the function of the aforementioned TCMs on adult neurogenesis. In addition, administering Scorpion venom heat resistant peptide (SVHRP) promotes astrocytes to release BDNF and promotes the growth of axons of immature neurons. However, blocking BDNF with anti-BDNF antibodies can eliminate these SVHRP-dependent neurotrophic effects (Wang et al., 2014).

In addition to regulating BNDF to enhance adult neurogenesis, TCM can also control other neurotrophic factors to enhance adult neurogenesis. In TBI rats, “Shengyu” decoction can increase the expression of glial cell line-derived neurotrophic factor (GDNF) and nerve growth factor (NGF) for the proliferation of NSCs (Chen M. M. et al., 2015). Huatuo Zaizao extract can boost the production of newly formed neurons, and increase the levels of VEGF and BDNF (Zheng et al., 2014), in addition to BDNF, NGF, TrkB, TrkA, which are all upregulated by *Xiaoyao pills* (Fang et al., 2020). Additionally, Angelica sinensis (Oliv.) Diels not only promotes the proliferation and maturation of hippocampal NSCs, but can also upregulate the expression of BDNF, GDNF, and vascular endothelial growth factor A (VEGF-A) in the hippocampus in chronic cerebral ischemia models (Cheng et al., 2021).

### 4.2. Increase of neurotransmitters

Depression is associated with decreased adult neurogenesis and abnormal monoammonia levels (Lanni et al., 2009; Jiang et al., 2022). Importantly, monoamine neurotransmitters function to increase neurogenesis (Cameron et al., 1998). A 5-Hydroxytryptamine (5-HT) reuptake inhibitor like fluoxetine not only has an obvious antidepressant effect but can also greatly improve adult neurogenesis in depression models. The antidepressant effects of Jie Yu Chu fan capsules in depressed mice can enhance adult neurogenesis by increasing the levels of norepinephrine (NE) and dopamine (DA) (Ji et al., 2020). Jieyu Anshen granules improving the neurological and cognitive functions of PSD model mice may be related to increases in the levels of NE, DA, and 5-HT (Du et al., 2020). Curcumin increased hippocampal adult neurogenesis, which may be related to curcumin increasing 5-HT (1a) mRNA in the hippocampal subregion after stress (Xu et al., 2007). The mechanism by which the above TCMs may influence neurogenesis by affecting neurotransmitters is shown in Figure 7B.

#### 4.2.1. Inflammation reduction

Pro-inflammatory factors IL-1β, IL-6, and NF-κB produced by activated microglia or astrocytes may impact different phases of adult neurogenesis (Ekdahl et al., 2009; Czeh et al., 2011). TCMs (Figure 7C) may alleviate abnormal adult neurogenesis by reducing glial cell activation and inflammatory factors.

One strategy for TCM to increase adult neurogenesis is to inhibit microglial activation. The influence of α-Asarone on neurogenesis may be correlated with a decline in the proportion of activated microglia, a reduction in microglial numbers, and the maintenance of velocity (Cai et al., 2016). Kososan extract can prevent the avoidance behavior of socially failed mice, which is partially mediated by the downregulation of hippocampal neuroinflammation, possibly through the regulation of increased anti-inflammatory microglia and adult hippocampal neurogenesis (Ito et al., 2017). Erinacine A and erinacine S promote hippocampal adult neurogenesis in AD mice, which may lessen glial cell activation (Tzeng et al., 2018).

Another strategy for TCM to increase adult neurogenesis at different stages is to inhibit the release of proinflammatory cytokines (TNF-α, IL-1β, and IL-6) by microglia. In the proliferation stage, ZTC increases NSC proliferation and inhibits the expression level of NF-kB in a dose-dependent manner (Yang et al., 2020). Under the differentiation stage, Ligusticum chuanxiong (LC) significantly increased DCX in the hippocampal DG of adult rats 14 days after cerebral ischemia. Meanwhile, LC reduces IL-1β and TNF-α (Wang et al., 2020). THSG, the main active compound of the traditional Chinese herb *Polygonum multiflorum*, can lower TNF-α, IL-1β, and IL-6 (Jiang et al., 2018). At the maturation stage, Xiaoyao pills increase newly formed neurons and significantly decrease the levels of IL-6 and TNF-α (Fang et al., 2020). Treatment with Polygoni multiflori radix extract can greatly increase the number of new neurons after an ischemic stroke. This may be accomplished by blocking the TLR4/NF-κB/NLRP3 inflammatory signaling pathway after an ischemic stroke in rats (Li et al., 2020). Moreover, Jieyu Anshen granules (Du et al., 2020), Hericium erinaceus mycelium (HEM), and an isolated diterpenoid derivative known as erinacine A (Tsai et al., 2019) all support the development of new neurons and can reduce TNF-α and IL-1 β, which are linked to the regulation of adult neurogenesis (Du et al., 2020).

Traditional Chinese medicine can inhibit both microglia activation and the release of proinflammatory factors. Andrographolide (Lu et al., 2019) inhibits chronic stress-induced abnormalities in adult hippocampal neurogenesis by reversing microglia-mediated pro-inflammatory cytokine production. Nuclear transcription factor NF-κB level decreased, and LPS-induced IL-1β level was changed by ALWPS-regulated FAK signal. Moreover, ALWPS significantly inhibited the LPS-induced migration of BV2 microglia. Oral administration of ALWPS to C57BL/6 J mice injected with LPS can greatly improve short- and long-term memory. More importantly, oral treatment of ALWPS significantly reduced microglia activation in the hippocampus and cortex (Lee et al., 2018).

Additionally, TCM may regulate astrocyte anti-inflammation and increase NSC proliferation. Ilexonin A can enhance NSC proliferation by activating astrocytes and decreasing TNF-α and IL-1 β (Xu et al., 2020). Ginsenoside Rg1 decreased astrocyte activation and increased hippocampal cell proliferation by reducing IL-1β, IL-6, and TNF-α (Zhu et al., 2014).

#### 4.2.2. Reduction of neurotoxicity

In the past few years, impaired adult hippocampal neurogenesis has emerged as a hallmark of AD pathophysiology along with Aβ and tau hyperphosphorylation-induced neurotoxicity, and further research has shown that Aβ-induced neurotoxicity is associated with altered neurogenesis and memory formation (Abshenas et al., 2020; Amber et al., 2020). Although Aβ causes a temporary increase in the number of neurons in younger mice, it also causes a drop in the NSC pool, which results in a lower rate of adult neurogenesis in older animals (Lopez-Toledano et al., 2010). In the existing animal model of AD, mice with Aβ intraperitoneal injections or transgenic Aβ accumulation can severely impair adult neurogenesis, and TCMs (Figure 7D) improved this situation. On the one hand, TCMs such as Cyperus rotundus extract (Shakerin et al., 2020), rosmarinic acid, and ursolic acid (Mirza et al., 2021) repaired the spatial memory damage induced by Aβ1-42 and increased adult neurogenesis. On the other hand, the transgene-induced aggregation of Aβ was also associated with an aberrant reduction of adult neurogenesis. In this situation, TCMs reduced Aβ deposition in the brain and enhanced hippocampal adult neurogenesis in AD animal models with different genetic backgrounds. For instance, Jowiseungchungtang inhibited the aggregation of Aβ and the pathology induced by Aβ in AD model mice (five family AD variants) and improved adult hippocampal adult neurogenesis *in vivo* (Shin et al., 2018), Icariin reduced Aβ in the brain of Tg2576 mice and enhanced adult hippocampal neurogenesis (Li et al., 2015), erinacine A and erinacine S inhibited the growth and reduced the load of Aβ plaque and promoted adult hippocampal neurogenesis in APPswe/PS1ΔE9 transgenic mice (Tzeng et al., 2018), and PM012 significantly reduced Aβ deposition and increased adult neurogenesis in 3xTG AD mice (Ye et al., 2016).

#### 4.2.3. Activation of PI3-AKT and ERK pathways

The ERK and PI3K / Akt pathways may regulate different stages of adult neurogenesis, including the growth, differentiation, maturation, and survival of NSCs (Shioda et al., 2009; Mellios et al., 2018). TCMs (Figure 7A) could affect adult neurogenesis by regulating the ERK and PI3K / Akt pathways of NSCs.

Salvianolic acid B maintains self-renewal and promotes the proliferation of NSCs *via* the PI3K / Akt signaling pathway, which is confirmed by PI3 (LY294002) inhibition eliminating this effect (Zhuang et al., 2012). Meanwhile, THSG (a primary active compound of the traditional Chinese herb *Polygonum multiflorum*) (Jiang et al., 2018) and the purified fraction of *Gastrodiae rhizoma* (Hsu et al., 2021) stimulate adult neurogenesis and regulate the PI3K/Akt pathway. Moreover, magnesium lithospermate B (Zhang Z. et al., 2018) and musk ketone (Zhou et al., 2020) promote the proliferation and differentiation of NSCs through the activation of the PI3K/Akt signaling pathway. Akti-1/2, an Akt inhibitor, also blocks the effect of musk ketone on NSCs. This suggests that Muscone promotes NSC proliferation and differentiation by activating the PI3K/Akt signaling pathway (Zhou et al., 2020). Chaihu Shugan San increases the levels of pPI3k/PI3K and pAkt/Akt in the hippocampus of stressed mice and restores the newly formed neurons. The two main active ingredients in Chaihu Shugan San, quercetin and luteolin, were then discovered to have a good docking fraction with the PI3K protein using molecular docking technology. This further confirmed that the PI3K/Akt pathway is how CSS participates in the treatment of MDD (Zhang et al., 2021).

Chaihu Shugan San not only boosted the PI3K/Akt pathway in stressed mice but also reduced the level of p-GSK3β/GSK3β to promote adult neurogenesis (Zhang et al., 2021). Danggui Jakyak San increases Akt/GSK3 β/β- Catenin signal transduction, which may be one of the mechanisms through which it promotes adult neurogenesis (Song et al., 2013). Similarly, raising p-Akt and p-GSK-3β is thought to play a factor in how alkaloids in Huanglian Jiedu decoction encourage NSC proliferation. Flavonoid treatment promotes the differentiation of cortical precursor cells into neurons rather than glial cells, which could be attributed to the upregulation of Akt and GSK-3β (Zou et al., 2016). In addition, Saikosaponin-d (SSD) inhibits cell viability and proliferation of hippocampal NPCs in a concentration-dependent manner. Subsequent research indicates that SSD suppresses adult neurogenesis and NPC proliferation *via* the GSK3 β/β- Catenin signaling pathway (Qin et al., 2019).

Traditional Chinese medicine could aid the PI3K/Akt/CREB pathway in NSC differentiation. Kami-ondam-tang greatly enhanced the expression of p-Akt and p-CREB in the hippocampal CA1 region and dentate gyrus, and, at the same time, the number of DCX-positive cells in the dentate gyrus increased significantly. These results suggest that Kami-ondam-tang improves cognitive ability by upregulating Akt/CREB/BDNF signaling and adult neurogenesis (Hong et al., 2011). Tetramethylpyrazine induces the release of BDNF from bone marrow mesenchymal stem cells by activating the PI3K/Akt/CREB pathway for neural differentiation. This effect could be reversed by the PI3K inhibitor LY294002 (Chen et al., 2021).

Traditional Chinese medicine could also influence NSC during development and survival *via* the Akt pathway. Baicalin induces neuronal development, matures them *via* the Akt/Foxg1 pathway, and sustains them to have an antidepressant effect (Zhang et al., 2019). By the activation of PI3K/Akt/BAD, Buyang Huanwu decoction (BHD) stimulates neurogenesis in apoptosis, proliferation, differentiation, maturation, and eventually the recovery of the function of learning and memory (Chen et al., 2020).

Another type of kinase that influences NSC proliferation, differentiation, and survival is extracellularly regulated protein kinases (ERK) (Rai et al., 2019). TCM promotes the proliferation of NSCs through ERK. Acorus tatarinowii and its components, α- asarone and β- asarone, promote NPC proliferation *in vitro*. Subsequent research has shown that Acorus tatarinowii and asarone activated ERK but did not activate the Akt pathway; FR180204 inhibited ERK activity and effectively blocked the promoting effect of Acorus tatarinowii or asarone on the proliferation of NPC (Mao et al., 2015). In contrast to Acorus tatarinowii, Sun ginseng increases p-ERK and p-Akt levels in addition to NSC proliferation and survival, which may be the method through which memory is enhanced (Lee et al., 2013). In addition, Zhengtian capsules promote the proliferation of hippocampal NSCs and the protein levels of phosphorylated ERK1/2 and CREB (Yang et al., 2020).

#### 4.2.4. Activation of the Notch-Hes pathway

Activation of the Notch signaling pathway enhances the production of Hes1 and Hes5, which promote stem cell proliferation and inhibit neuronal differentiation (Mendes-da-Silva et al., 2015; Zhang R. et al., 2018; Ohtsuka and Kageyama, 2021). TCM may influence adult neurogenesis by regulating stem cell proliferation and differentiation *via* the Notch1/Hes pathway (Figure 7E). Zhuang et al. (2012) screened 45 bioactive components from TCM, which were widely used in the treatment of stroke in China, and evaluated their effect on the proliferation of neural stem/progenitor cells. The results showed that Sal-b promoted NSC self-renewal along with an increase in Notch1 gene expression. The Buyang Huanwu decoction increased the expression of Hes1 and promoted NSCs to differentiate into astrocytes (Chen et al., 2020). More importantly, TCMs regulate neurogenesis under different pathological conditions through the Notch1/Hes5 pathway, which may have a time effect from the Yi-nao-jie-yu prescription (Tian et al., 2018) and a dosage effect from the Ginseng-Angelica-Sansheng-pulvis combination (Liu et al., 2019).

## 5. Toxic and side effects

There are only a few clinical reports on the toxicity and adverse effects of TCMs regulating adult neurogenesis, whether used alone or in combination. The side effects of TCMs, including MLC901 (Kumar et al., 2020), curcumin (Asher and Spelman, 2013; Fan et al., 2013), and Polygala tenuifolia (Zhao X. et al., 2020), are largely gastrointestinal, such as nausea, vomiting, and diarrhea. The majority of side effects are mild and temporary, and after discontinuing the medication, these symptoms will gradually subside. There are also a few reports on the side effects of TCMs in other systems. Pseudoaldosteronism caused by Yokukansan (Ishida et al., 2020; Katsuki et al., 2021) causes hypertension, hypokalemia, and muscular weakness, which may lead to death. Therefore, patients must be aware of the risks when considering taking Yokukansan (Ishida et al., 2020). Curcumin may chelate dietary trace elements, and long-term supplementation of curcumin aggravates iron deficiency (Chin et al., 2014). Clinicians should pay attention to any side effects that could increase the number and function of myeloid-derived suppressor cells when using angelica polysaccharide as an immune enhancer (Shen et al., 2022). Cornus officinalis extract has shown good results in treating drug-resistant asthma, but it may cause allergic contact dermatitis (Mirsadraee et al., 2018).

The toxic and adverse effects of combining TCMs with Western medicine have also been documented and require special attention. TCMs may affect the activity of the cytochrome P450 (CYP) enzyme system, which may enhance therapeutic effects but could also lead to increased side effects. For the treatment of epilepsy, Gastrodiae rhizoma might lengthen the plasma half-life and concentration of carbamazepine and its metabolite (carbamazepine-10, 11-epoxide). However, it could also be accompanied by an expansion of the neurological signs of toxicity (Yip et al., 2020). Ginkgo stimulates both CYP3A4 and CYP2C9 and alters the AUC and Cmax of conventional medications like midazolam, tolbutamide, lopinavir, and nifedipine. Ginsenosides Re increased CYP2C9, which reduced the anticoagulant activity of warfarin (Suroowan and Mahomoodally, 2019). In addition, Glycyrrhizae radix et rhizoma replaces serum-bound cardiovascular medications and reduces the disease-treating effects of diltiazem, nifedipine, and verapamil (Suroowan and Mahomoodally, 2019). Individuals who took ginger and aprepitant together experienced more severe acute nausea than those who took only aprepitant (Zick et al., 2009). Despite the limited and contradictory results about curcumin enhancing the function of doxorubicin-induced cardiac toxicity, it is necessary to conduct carefully designed research to evaluate the safety and effectiveness of the new formulation of this compound during cancer treatment (Armandeh et al., 2022).

It should be noted that ingesting an excessive amount of TCMs, even “medicine food homologous,” will produce adverse reactions. For example, Korean red ginseng (KRG) is very popular as a dietary supplement, but its excessive intake can cause “shanghuo,” which is closely related to the acceleration of the TCA cycle and the increase of AMPK activity (Zhao T. et al., 2020). At a regular dose, Morinda officinalis has not been associated with any significant negative effects in clinical trials, but in some cases, doses greater than 1 g/kg have been linked to irritability, insomnia, and unpleasant feelings (Zhang J. H. et al., 2018). Excessive intake of curcumin may have adverse effects on the kidney, heart, liver, blood, and immune system, which is a reminder that there is still much research to be done before curcumin can be effectively used and transformed (Liu et al., 2022). High doses of baicalin improve the antioxidant system in rat liver, but at the same time, they also lead to the reduction of trace minerals, thereby decreasing the activity of some metal-containing enzymes and having negative health implications (Gao et al., 2003).

Based on the aforementioned reports, TCMs that regulate adult neurogenesis should be used with caution in clinical applications due to their toxicity and side effects. Regarding the effectiveness, toxicity, and side effects of TCMs on adult neurogenesis, quality control and reliability of TCMs are also important determining factors. Genuine traditional Chinese materia medica, processing, safety, compatibility with other medications, and dosage of TCMs used for different medical conditions should all be taken into consideration. It is also necessary to keep researching the scientific and ethical principles of TCM clinical trials on adult neurogenesis. All of these techniques can effectively protect the subjects’ rights, interests, and safety while also improving the development of TCMs on adult neurogenesis to prevent and treat nervous system disorders.

## 6. Conclusion and future work

Many studies have shown that adult neurogenesis plays an important role in the regulation of neurological and psychotic disorders. A better understanding of the effects and mechanisms that regulate adult neurogenesis will identify disease pathologies that drive cognitive and emotional impairments, thereby providing an avenue for the development of effective therapeutic strategies. Here, we have focused on the role of adult neurogenesis in neuropsychiatric disorders, especially the characteristics and mechanisms of the ameliorative effects of TCM resulting from its regulation of adult neurogenesis. This review provides recent evidence on the regulation of adult neurogenesis by TCM.

Extensive studies have made significant progress in the regulation of adult neurogenesis thanks to TCM, but there are still many questions and thus further studies are needed. (1) Although adult neurogenesis has been shown to exist in animals, there is insufficient evidence to date to adequately support its existence in adult humans. It is crucial for future research to explore the dynamic changes and the functional role of adult neurogenesis in the normal human brain and alterations in neuropsychiatric disorders. More accurate approaches, cell markers, and human imaging protocols that can efficiently study adult neurogenesis are the greatest necessities in this field. (2) Although many promising results have been achieved by using TCM to regulate adult neurogenesis in various animal models and in *in vitro* cell cultures, no clinical trials have been conducted so far. One limitation that hinders the clinical trials of drugs on adult neurogenesis is the lack of an *in situ* method to monitor and calculate adult neurogenesis. However, greater efforts should be made to conduct clinical research to further verify the efficacy of TCM in improving adult neurogenesis in humans. (3) The discovery of effective ingredients from TCM to improve adult neurogenesis holds great promise, but current studies on the exact targets and the pathways involved are far from sufficient. The study of the exact pharmacological targets of TCM for improving adult neurogenesis should be further conducted in the future. With the aid of new methods such as bioinformatics, it would be clarified, and then more effective agents could be designed and developed accordingly.

Although adult neurogenesis *per se* has not yet yielded a clinically approved compound for any indication, the target remains of interest and is under investigation for drug development. TCM is a great treasure that provides abundant sources for drug discovery to modulate adult neurogenesis. We believe that the future development of medications from TCM that can improve adult neurogenesis would bring us one step closer to its application in the treatment of human diseases.

## Author contributions

WS reviewed the databases and analyzed the information on subjects. NJ and WZ designed this review and worked on the manuscript revision. WS and NJ wrote the draft and modified this article. WZ revised this article and replied to the reviewers in the modification phase. All authors contributed to the article and approved the submitted version.

## Funding

This work was supported by the National Key R&D Program of China (Grant No. 2022YFC3500304).

## Conflict of interest

The authors declare that the research was conducted in the absence of any commercial or financial relationships that could be construed as a potential conflict of interest.

## Publisher’s note

All claims expressed in this article are solely those of the authors and do not necessarily represent those of their affiliated organizations, or those of the publisher, the editors and the reviewers. Any product that may be evaluated in this article, or claim that may be made by its manufacturer, is not guaranteed or endorsed by the publisher.
